# Generalization and Expansion of the Hermia Model for a Better Understanding of Membrane Fouling

**DOI:** 10.3390/membranes13030290

**Published:** 2023-02-28

**Authors:** Gustavo Leite Dias Pereira, Lucio Cardozo-Filho, Veeriah Jegatheesan, Reginaldo Guirardello

**Affiliations:** 1 Department of Chemical Engineering, State University of Maringa, Maringa 87020-900, Brazil; 2 School of Engineering and Water, Effective Technologies and Tools (WETT) Research Centre, RMIT University, Melbourne, VIC 3000, Australia; jega.jegatheesan@rmit.edu.au; 3 College of Chemical Engineering, State University of Campinas, Campinas 13083-852, Brazil

**Keywords:** membrane fouling, Hermia model, fouling model, pore blocking, blocking mechanism

## Abstract

One of the most broadly used models for membrane fouling is the Hermia model (HM), which separates this phenomenon into four blocking mechanisms, each with an associated parameter n. The original model is given by an Ordinary Differential Equation (ODE) dependent on n. This ODE is solved only for these four values of n, which limits the effectiveness of the model when adjusted to experimental data. This paper aims extend the original Hermia model to new values of n by slightly increasing the complexity of the HM while keeping it as simple as possible. The extended Hermia model (EHM) is given by a power law for any *n* ≠ 2 and by an exponential function at *n* = 2. Analytical expressions for the fouling layer thickness and the accumulated volume are also obtained. To better test the model, we perform model fitting of the EHM and compare its performance to the original four pore-blocking mechanisms in six micro- and ultrafiltration examples. In all examples, the EHM performs consistently better than the four original pore-blocking mechanisms. Changes in the blocking mechanisms concerning transmembrane pressure (TMP), crossflow rate (CFR), crossflow velocity (CFV), membrane composition, and pretreatments are also discussed.

## 1. Introduction

**Figure 1:**
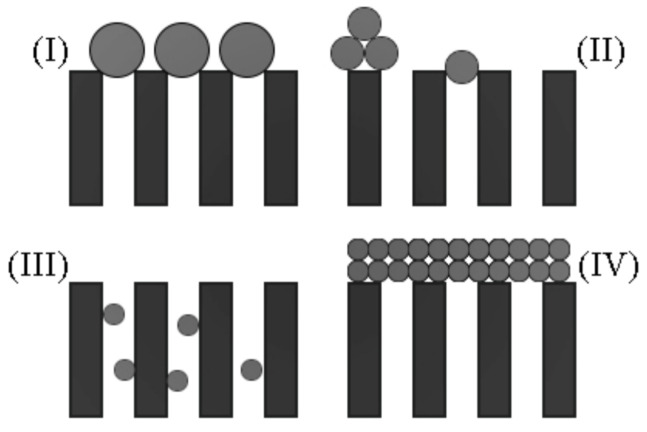
Blocking mechanisms by Hermia (1982): (**I**) complete blocking; (**II**) intermediate blocking; (**III**) standard blocking; (**IV**) cake formation.

One of the most widely used models to predict membrane fouling is the Hermia model (HM) [1]. In a 1982 paper, Hermia was able to frame mathematically the relationship between the accumulated volume and time from experimental data, arriving at the differential equation presented in Equation (1) [1]. Since this model was derived for non-Newtonian fluids, the parameters n and k help to adjust the model for different types of fluids and blocking mechanisms. The original ordinary differential equation (ODE) was solved for four different discrete values of n, each value with its blocking mechanism, as shown in Figure 1 and Equations (2)–(5).

(1) $$\frac{d^{2}t}{dV^{2}}=k{\left(\frac{dt}{dV}\right)}^{n}$$

(2) $$\mathrm{Complete}\;\mathrm{blocking}\;(\mathrm{CB})\;(n=2)\;\ln J=\ln J_{0}-k\cdot t$$

(3) $$\mathrm{Intermediate}\;\mathrm{blocking}\;(\mathrm{IB})\;(n=1)\;1/J=\left(1/J_{0}\right)-k\cdot t$$

(4) $$\mathrm{Standard}\;\mathrm{blocking}\;(\mathrm{SB})\;(n=3/2)\;1/\surd J=\left(1/\surd J_{0}\right)-k\cdot t$$

(5) $$\mathrm{Cake}\;\mathrm{formation}\;(\mathrm{CF})\;(n=0)\;1/J^{2}=\left(1/J_{0}^{2}\right)-k\cdot t$$

where t is the time measured from the beginning of the filtration experiment, j is the permeate flux at time t, $j_{0}$ is the permeate flux at time t=0, and k is a real constant determined experimentally. Due to its simplicity, the Hermia model has been applied in many areas with varying degrees of success (Table 1), such as in the filtration of polyethylene glycol [2], glycerin-water solutions [3], oily effluent [4], microalgae [5], organic matter [6], and polycyclic hydrocarbons [7]. Other uses include the modeling of fouling mechanisms in biofilm-membrane bioreactors [8], as well as use in combined pore-blocking mechanism models [9].

As with any numerical model, the HM can have varying performances, depending on the data obtained during experiments. This can be a result of many different factors, such as the experimental setup, measurement quality, interactions between the membrane and the fluid in question, and changes to the membrane’s surface due to successive fouling layers. As an example, high performance can be obtained for more than one pore-blocking mechanism, such as in [7], in which a nanofiltration membrane NF10 obtained correlation coefficients of 0.991 for SB and 0.988 for IB. Similarly, another nanofiltration membrane NF270 obtained correlation coefficients of 0.947 for SB and 0.936 for IB. This behavior can also be found in untreated effluent [6], in which an ultrafiltration membrane obtained R^2^ coefficients of 0.994 and 0.952 for SB and CF, respectively. As a result, if the experimental setup and data collection are properly carried out, more than one pore-blocking mechanism can be numerically representative, which makes it difficult to determine which mechanism is more prevalent.

In some other cases, assuming that the experimental results are sufficiently accurate, it is also possible that none of the four classic mechanisms adjusts well. One example of this can be found in the ultrafiltration of polyethylene glycol [2], in which for TMPs below 0.2 MPa, all blocking mechanisms obtained correlation coefficients smaller than 0.813 for all crossflow velocities (CFVs) tested. Similar results have also been reported in water-glycerin solutions [3], in which the membranes PES25 and PVDF only obtained R^2^ coefficients below 0.870 for a triglyceride-water solution. There are also cases in which one blocking mechanism performs better than the rest, such as in the filtration of albumin solutions [10], in which CF was the best-performing pore-blocking mechanism for all tests conducted.

As a result, for some applications the HM can be numerically representative and help determine the most prevalent blocking mechanism; however, even with accurate experimental data, there are cases where the HM is neither numerically representative nor helpful for pore-blocking analysis. Thus, many authors have employed the use of more complex and nuanced models [11,12], such as the Arnot model [13,14] expressed in Equation (6).

(6) $$J=J_{0}{\left[1+k\left(n-2\right){\left(AJ_{0}\right)}^{2-n}t\right]}^{\frac{1}{\left(n-2\right)}}$$

For n=1.5, 1.0, and 0. These values of n correspond to the three pore-blocking mechanisms SB, IB, and CF, respectively. This model was used by Pan et al. [14] to analyze the membrane resistance and how the controlling stages change concerning TMPs between 0.10 and 0.14 MPa and CFRs of 25 to 60 L/h. Since the Arnot model is a reformulation of the HM, some of its qualities and setbacks are also found in this model.

Other examples of fouling models can be found in reverse osmosis desalination [12], in the form of water permeability coefficient-based models. In this model class, equations are used to estimate the decline of the permeate flux over time due to variations in the water permeability coefficient A. More specifically, these models have the goal of estimating the normalized water permeability coefficient $A_{n}$. The simplest equation is given by the Wilf model (Equation (7)) [15].

(7) $$A_{n}=t^{m}$$

where t is time given in days and m is a real number between −0.035 and −0.041 [15]. Similar to the Wilf model, other authors also modeled $A_{n}$ with more complex equations based on the exponential function [16,17,18]. The Zhu, Abbas, and Ruiz-García models, Equation (8), Equation (9), and Equation (10), respectively, denote an increase in the degrees of freedom to better accommodate experimental data.

(8) $$A_{n}=A_{0}\exp\left(-\frac{t}{\tau }\right)$$

(9) $$A_{n}=\alpha \exp\left(\frac{\beta }{t+\gamma }\right)$$

(10) $$A_{n}=\delta_{1}\exp\left(-\frac{t}{\tau_{1}}\cdot k_{fp}\right)+\delta_{1}\exp\left(-\frac{t}{\tau_{1}}\cdot k_{fp}\right)$$

where the Greek letters τ, β, γ, and δ, as well as $k_{fv}$, are determined based on experimental results [12]. Although these models have been used and have a good performance [15,17,18,19,20], one of the major setbacks is the need for long-term operating data, which is not always available [12]. For systems that tend to a constant permeate flux, the Mondal-De model (Equation (11)) presents a simple equation that has two dimensionless constants $A_{1}$ and $A_{2}$, making it possible to obtain a good model fitting [21].

(11) $$\frac{J_{SS}}{J_{0}}=\frac{1}{\left(1+A_{1}\right)\left(1+A_{2}\right)}$$

In a 1993 paper, Field and collaborators reported interesting fouling behavior in cross-flow microfiltration [22]. Given the results published by the group, experiments seem to show that it is possible to operate a microfiltration membrane at a constant flux without any increase in TMP; therefore, the team concluded that fouling was slight or negligible at lower pressures. It was also shown that an increase in TMP is followed by fouling and flux reduction. Thus, Field et al. formulated the critical flux hypothesis for microfiltration, which states that on start-up, there is a flux below which flux decline does not happen. Above this flux, fouling can be observed. As a result, there is a sort of critical flux $j^{*}$ that seems to act like a tipping point for fouling. Given this context, it is possible to incorporate this concept into Hermia’s equations, which results in the critical flux model (Equation (12)).

(12) $$-\frac{dJ}{dt}\left(J^{n-2}\right)=k\left(J-J^{*}\right)$$

where n is the same blocking mechanism parameter from the Hermia model. In Equation (12), n can assume the values of 0, 1, and 2, which correspond to CF, IB, and CB, respectively. It is also possible to express the pore-blocking mechanisms in terms of the accumulated volume. As an example, even though the Hermia model was developed for dead-end filtration, Khan et al. [9] developed a participation equation for cross-flow filtration (Equation (13)) that builds upon the works of Hermia [1], Sampath et al. [23] (Equations (14)–(17)), Kilduff et al. [24] (Equations (18)–(20)), Bowen et al. [25] (Equations (21)–(24)), and Wiesner et al. [26] ((Equations (25)–(28)). These models are modified versions of the four pore-blocking mechanisms. The participation equation has one constant β for every blocking mechanism, such that the accumulated volume V is the sum of all accumulated volumes for all mechanisms.

(13) $$V=\beta_{b}V_{b}+\beta_{i}V_{i}+\beta_{s}V_{s}+\beta_{gl}V_{gl}$$

The accumulated volumes $V_{b}$, $V_{i}$, $V_{s}$, and $V_{gl}$ are calculated based on the fouling models obtained by previous publications. Equations (14)–(17) are adaptations of the fouling models obtained by Sampath et al. [23] for cross-flow filtration.

(14) $$\mathrm{Complete}\;\mathrm{blocking}\;(\mathrm{CB})\;V_{b}=\frac{A_{0}J_{0}}{K_{b}}\left(1-e^{-K_{b}t}\right)$$

(15) $$\mathrm{Intermediate}\;\mathrm{blocking}\;(\mathrm{IB})\;V_{i}=\frac{A_{0}\ln\left(1+K_{i}J_{0}t\right)}{K_{i}}$$

(16) $$\mathrm{Standard}\;\mathrm{blocking}\;(\mathrm{SB})\;V_{s}=A_{0}{\left(\frac{1}{J_{0}t}+\frac{K_{s}}{2}\right)}^{-1}$$

(17) $$\mathrm{Cake}\;\mathrm{formation}\;(\mathrm{CF})\;V_{gl}=\frac{A_{0}}{K_{gl}J_{0}}(\sqrt{1+2K_{gl}J_{0}^{2}t}-1)$$

Similarly, Equations (18)–(20) are the fouling models obtained by Kilduff et al. [24] in terms of the accumulated volume for cross-flow filtration.

(18) $$\mathrm{Complete}\;\mathrm{blocking}\;(\mathrm{CB})\;V_{b}=\frac{A_{0}\left(1-e^{-J_{0}K_{b}t}\right)}{K_{b}}$$

(19) $$\mathrm{Complete}\;\mathrm{blocking}\;(\mathrm{CB})\;V_{b}=\frac{A_{0}\left(1-e^{-J_{0}K_{b}t}\right)}{K_{b}}$$

(20) $$\mathrm{Cake}\;\mathrm{formation}\;(\mathrm{CF})\;V_{gl}=\frac{\sqrt{1+4K_{gl}J_{0}A_{0}^{2}}-1}{2K_{gl}A_{0}}$$

Khan et al. [9] also applied the same participation equation with the fouling models by Bowen et al. [25], resulting in Equations (21)–(24) for cross-flow filtration.

(21) $$\mathrm{Complete}\;\mathrm{blocking}\;(\mathrm{CB})\;V_{b}=\frac{J_{0}}{K_{b}}\left(1-e^{-K_{b}t}\right)$$

(22) $$\mathrm{Intermediate}\;\mathrm{blocking}\;(\mathrm{IB})\;V_{i}=\frac{J_{0}\ln\left(1+K_{i}t\right)}{K_{i}}$$

(23) $$\mathrm{Standard}\;\mathrm{blocking}\;(\mathrm{SB})\;V_{s}=\frac{J_{0}t}{1+K_{s}t}$$

(24) $$\mathrm{Cake}\;\mathrm{formation}\;(\mathrm{CF})\;V_{gl}=\frac{2J_{0}}{K_{gl}}\left(\sqrt{1+K_{gl}t}-1\right)$$

Furthermore, the same treatment for cross-flow filtration was applied to the fouling models published by Wiesner et al. [26], in the form of Equations (25)–(28)

(25) $$\mathrm{Complete}\;\mathrm{blocking}\;(\mathrm{CB})\;V_{b}=A_{0}tJ_{0}\left(1-e^{-K_{b}t}\right)$$

(26) $$\mathrm{Intermediate}\;\mathrm{blocking}\;(\mathrm{IB})\;V_{i}=\frac{A_{0}tJ_{0}}{1+J_{0}K_{i}t}$$

(27) $$\mathrm{Standard}\;\mathrm{blocking}\;(\mathrm{SB})\;V_{s}=A_{0}t{\left(\frac{J_{0}^{\frac{1}{2}}}{1+J_{0}^{\frac{1}{2}}K_{s}t}\right)}^{2}$$

(28) $$\mathrm{Cake}\;\mathrm{formation}\;(\mathrm{CF})\;V_{gl}=A_{0}t\left(\sqrt{\frac{J_{0}^{2}}{1+J_{0}^{2}K_{gl}t}}\right)$$

With this model, Khan et al. [9] were able to obtain representative values of R^2^ for the removal of organic matter in water treatment. One of the setbacks of this model is its complexity when compared to other models. In a paper by Jegatheesan et al. [27], the models of cake filtration (Equation (29)), pore narrowing (Equation (30)), and a combination of external and internal progressive internal fouling (Equation (31)) were used in the modeling of fouling for the treatment of limed and partially clarified sugarcane juice.

(29) $$\frac{t}{V_{f}}=\frac{1}{Q_{0}}+\frac{\alpha C_{w}V_{f}}{2A_{0}R_{m0}Q_{0}}$$

(30) $$\frac{1}{Q_{f}^{0.5}}=\frac{1}{Q_{0}^{0.5}}+\frac{CQ_{0}^{0.5}t}{V_{p}}$$

(31) $$\frac{1}{Q_{f}}=\left[\frac{\mu_{f}}{P_{tm}A_{0}}\right]\left(\frac{\alpha C_{W}}{A_{0}}+\frac{2C}{V_{p}}\right)V_{f}+\left[\frac{\mu_{f}R_{m0}}{P_{tm}A_{0}}\right]$$

In the context of this study, the authors obtained correlation coefficients above 0.9718 for all three models at a TMP of 1 bar and a CFV of 3 m/s [27].

Given the models presented thus far, it is interesting to point out that both simple and complex models can have good numerical performance. Still, some models do not have the complexity to completely represent fouling behavior, while other modified models can have mixed results concerning numerical representativity, as in the work published by Bowen et al. [25]. Although enough complexity is needed, it is also important to have readily available equations that can be easily applied, such as shown with the Arnot and Mondal-De models. Therefore, the present paper aims to add some complexity to the original HM by unifying Equations (2)–(5) into a set of two equations that can be used to represent more values of n. We also aim to test this extended Hermia model (EHM) in data already available in the literature and compare its performance to the original Hermia model.

## 2. Materials and Methods

### 2.1. Control Volume and Model Setup

**Figure 2:**
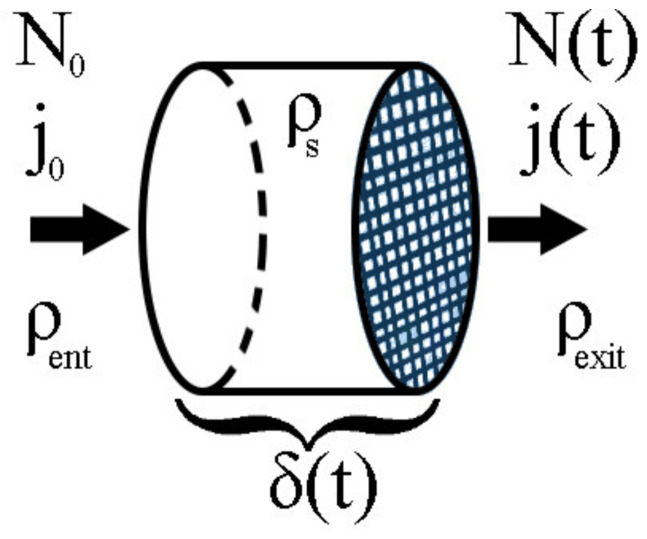
Control volume used in the proofs of Theorems 1–3.

Inside of a module (Figure 2) of constant cross-sectional area A and density $\rho_{s}$, the entering stream of fluid has a constant mass flux $N_{0}$, a constant permeate flux $j_{0},$ and density $\rho_{ent}$. The exiting stream of fluid has a mass flux $N\left(t\right)$, a permeate flux $j\left(t\right),$ and a density $\rho_{exit}$. As a result, some mass from this flux will be retained by the membrane, making it harder for more mass to pass through the membrane as permeate. Consequently, over time, the exit mass flux $N\left(t\right)$ should decrease. In this model, the mass accumulated is modeled by a porous solid with a constant base area A and thickness $\delta \left(t\right)$. After an infinitesimal time Δt, the mass accumulated results in an increase in δ. Therefore, it is possible to take this solid as the control volume (CV) and apply conservation laws to it.

### 2.2. Continuity Equation

The continuity equation (Equation (5)), also known as the general conservation of mass equation [5] or as the mass balance equation [28,29], is the mathematical formula that keeps track of how much mass is inside a given control volume. It does so by computing how much the control volume itself changes over time, which is given by the volume integral, and by calculating how much mass leaves or enters the CV, which is given by the surface integral.

(32) $$\frac{\partial }{\partial t}\underset{CV}{∭}\rho d\forall +\underset{CS}{\iint }\rho (\vec{j}\cdot \vec{n})dA=0$$

where ρ is the density function, d∀ is the volume differential of the CV, $\vec{j}$ is the velocity vector, $\vec{n}$ is the vector perpendicular to the surface of the CV, and dA is the differential surface area of the CV.

### 2.3. Hermia’s Experimental Model

The differential equation (Equation (1) or Equation (33)) is an experimental result obtained by Hermia [1], which correlates the second derivative of time (t) concerning the accumulated volume (V) with the first derivative of t with respect to V.

(33) $$\frac{d^{2}t}{dV^{2}}=k{\left(\frac{dt}{dV}\right)}^{n}$$

Here, the coefficients k and n are two real numbers that can be changed to better adjust the model for different situations. As discussed in Section 1, the model was originally solved for n=2, 1, 3/2, 0. These solutions resulted in Equations (2), (3), (4), and (5), respectively.

### 2.4. Derivatives of Inverse Functions

For a given function $y\left(x\right)$ and its inverse function given by $x\left(y\right)$, the relationship between dy/dx and dx/dy, if $y\left(a\right)=b$, is given by [30]:

(34) $${\left(\frac{dy}{dx}\right)}_{x=a}\cdot {\left(\frac{dx}{dy}\right)}_{y=b}=1$$

As for the second derivatives of these functions:

(35) $${\left(\frac{d^{2}y}{dx^{2}}\right)}_{x=a}=-{\left(\frac{d^{2}x}{dy^{2}}\right)}_{y=b}\cdot {\left[{\left(\frac{dy}{dx}\right)}_{x=a}\right]}^{3}$$

For the sake of brevity, the proof does not use the full subscript (e.g., x=a). It only discloses the direct values of the independent variables. As an example, ${\left(dy/dx\right)}_{x=a}\;$ would be written as ${\left(dy/dx\right)}_{a}$.

### 2.5. Flux Definition

One of the definitions of mass flux of a given stream i is given by the product between its mass concentration/total density ($\rho_{i}$) and its velocity ($j_{i}$) [28,29], as stated in Equation (36).

(36) $$N_{i}=\rho_{i}j_{i}$$

### 2.6. Accumulated Volume and Flux

As presented in Equation (37), the accumulated volume $V\left(t\right)$ for a mass flux $N\left(t\right)$ can be calculated by integrating $N\left(t\right)$ from t = 0 to t = t, which will give the total mass per unit of cross-sectional area. Therefore, multiplying by the area and dividing by its density will yield the accumulated volume, $V\left(t\right)$.

(37) $$V\left(t\right)=\frac{A}{\rho_{exit}}\int_{0}^{t}N\left(t\right)dt$$

### 2.7. Integral Properties

A reduced form of the Leibnitz formula with constant integration limits a and b for a given function $y\left(x\right)$ yields Equation (38) [31].

(38) $$\overset{b}{\underset{a}{\int }}\left[\frac{dy\left(x\right)}{dx}\right]dx=y\left(b\right)-y\left(a\right)$$

## 3. Results

Taking into account Equations (1)–(4), it is possible to observe that, apart from n≠2, $j\left(t\right)$ seems to follow a pattern, such that, if the reduced permeate flux ($j/j_{0}$) is isolated in Equations (2)–(4):

(39) $$\mathrm{Intermediate}\;\mathrm{blocking}\;(n=1)\;j/j_{0}=1/\left(1-k\cdot t\cdot j_{0}\right)$$

(40) $$\mathrm{Standard}\;\mathrm{blocking}\;(n=3/2)\;{\left(j/j_{0}\right)}^{1/2}\;=1/\left(1-k\cdot t\cdot \surd j_{0}\right)$$

(41) $$\mathrm{Cake}\;\mathrm{formation}\;(n=0)\;{\left(j/j_{0}\right)}^{2}\;=1/\left(1-k\cdot t\cdot j_{0}^{2}\right)$$

Since k is a real number and $j_{0}$ is a constant, Equations (12)–(14) can be rewritten as one equation (Equation (42)) with a variable exponent P, where P≠0.

(42) $${\left[\frac{j\left(t\right)}{j_{0}}\right]}^{P}=\frac{1}{\left(1+k\cdot t\right)}$$

In this context, the pore-blocking mechanisms would be given by different values of P, such that P=1 is intermediate blocking, P=1/2 is standard blocking, and P=2 is cake formation. Furthermore, it is possible to establish a relationship between P and n, such that P=2−n. For n=2 (or P=0), the reduced permeate flux is simply given by Equation (43).

(43) $$\frac{j\left(t\right)}{j_{0}}=\exp\left(-k\cdot t\right)$$

We questioned wondered if other values of P can be used in Equation (42) to better represent experimental data, expanding the original model into a sort of extended Hermia model (EHM). Therefore, we performed the model fitting for all four original pore-blocking mechanisms and the EHM in Examples 1–6 to have a better understanding of how these mechanisms change in different contexts. In these Examples, we obtained consistently better performance than the four original pore-blocking mechanisms. Thus, to justify the use of the EHM, we also used Equations (5)–(11) and proven Theorems 1–3. Their proofs can be found in Appendix A, Appendix B, and Appendix C, respectively.

**Theorem** **1.** *The original Hermia model can be extended to accommodate new values of P. If both the fluid and the permeate have similar densities, then the flux*$j\left(t\right)$*can be expressed by Equation (44) for any*P≠0*. If*P=0*, then then the flux*$j\left(t\right)$*can be expressed by Equation (45).*

(44) $${\left[\frac{j\left(t\right)}{j_{0}}\right]}^{P}\approx \frac{1}{\left(1+k\cdot t\right)}\;$$

(45) $$j\left(t\right)\approx j_{0}\exp\left(-k\cdot t\right)$$

*A measure of how fast*$j\left(t\right)$*declines over time can be given by applying both Equations (44) and (45) and calculating the amount of time needed for the reduced permeate flux to drop by half*$\left(\frac{j\left(t\right)}{j_{0}}=0.5\right)$*. We refer to this quantity as the EHM half-life (Equation (46)).*

(46) $$t_{1/2}=\left\{\begin{array}{c} \frac{\left(\frac{1}{0.5^{P}}-1\right)}{k}\;,\;\;P\neq 0\\ -\frac{\ln0.5\;}{k},\;\;P=0 \end{array}\right.\;$$

*Therefore, for a given* P*, there is a p^th^-degree blocking mechanism. This means intermediate blocking is a 1st-degree blocking mechanism, that cake formation is a 2nd-degree blocking mechanism, and so on.*

**Theorem** **2.** *If the EHM has been correctly fitted to experimental data and represents the dataset well (such as with a low RMSE or with a high R^2^), then the fouling layer’s thickness can also be fitted to the profile given by Equation (47).*

(47) δt=k15+k16t−1P+1−k15−1P+1+k12t,  P≠0 k19exp−k9t−1+k20t,  P=0

**Theorem** **3.** *If the EHM has been correctly fitted to experimental data and represents the dataset well (such as with a low RMSE or with a high R^2^), then the accumulated permeate volume can be calculated using Equation (48).*

(48) $$V\left(t\right)=\left\{\begin{array}{c} \frac{j_{0}\cdot A}{k\left(1-\frac{1}{P}\right)}({\left[1+k\cdot t\right]}^{\left(1-\frac{1}{P}\right)}-1)\;,\;\;P\neq 0\\ \frac{j_{0}\cdot A}{k}\left[1-\exp\left(-k\cdot t\right)\right],\;\;P=0\; \end{array}\right.\;$$

**Example** **1.** *Model fitting for ultrafiltration membrane used in different wastewater pretreatment conditions.*

In a paper by Jung and Son, a pretreatment of organic matter coagulation and MIEX^®^ was evaluated on a bench-scale filtration apparatus. This work investigated many different pretreatment conditions and their impact on micro- and ultrafiltration in hydrophilic (HPI) and hydrophobic (HPO) membranes. While keeping TMP at 1 bar for microfiltration and 2 bar for ultrafiltration, both coagulant and MIEX^®^ were added to the wastewater, and the filtration was carried out [32].

**Figure A1:**
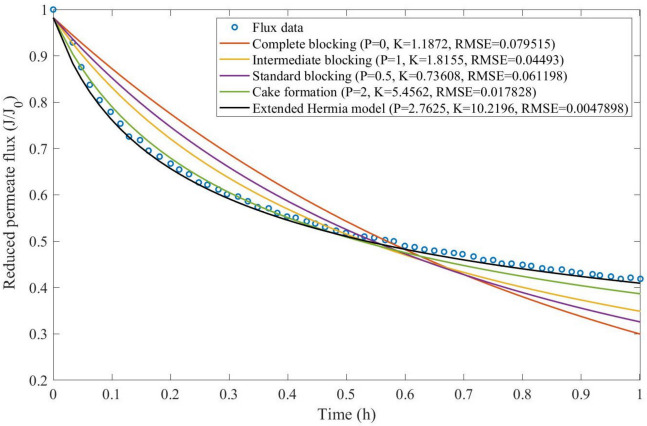
Model fitting for HPI ultrafiltration considering the four original pore-blocking mechanisms and the extended Hermia model (14).

**Figure A2:**
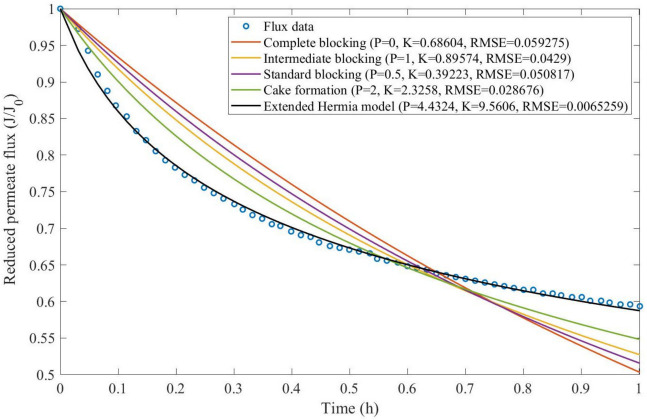
Model fitting for Coagulant 140 mg/L + HPI ultrafiltration considering the four original pore-blocking mechanisms and the extended Hermia model (14).

**Figure A3:**
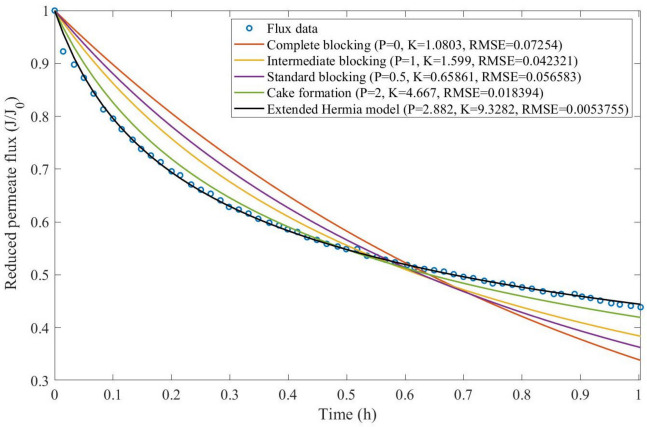
Model fitting for MIEX 12 mL/L + HPI ultrafiltration considering the four original pore-blocking mechanisms and the extended Hermia model (14).

**Figure A4:**
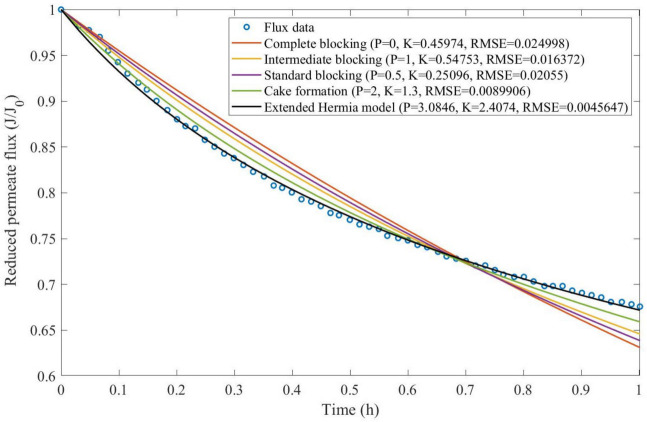
Model fitting for MIEX 12 mL/L + Coagulant 40 mg/L + HPI ultrafiltration considering the four original pore-blocking mechanisms and the extended Hermia model (14).

**Figure A5:**
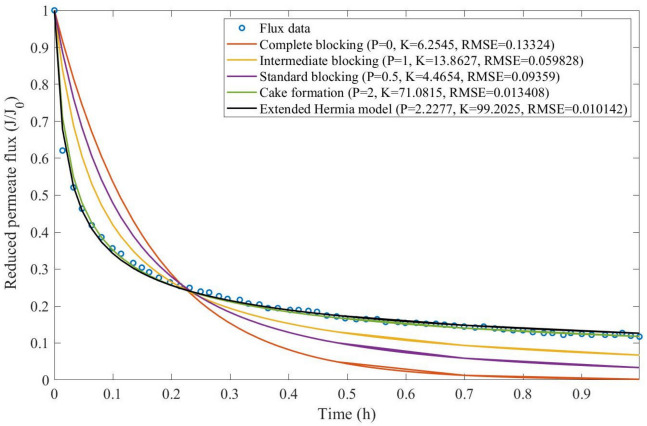
Model fitting for HPO ultrafiltration considering the four original pore-blocking mechanisms and the extended Hermia model (14).

**Figure A6:**
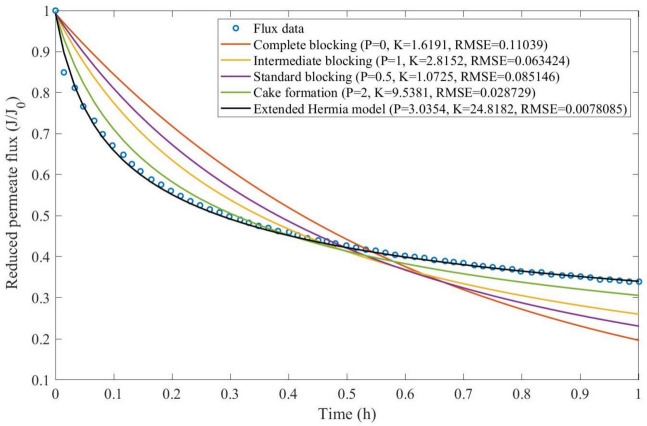
Model fitting for Coagulant 140 mg/L + HPO ultrafiltration considering the four original pore-blocking mechanisms and the extended Hermia model (14).

**Figure A7:**
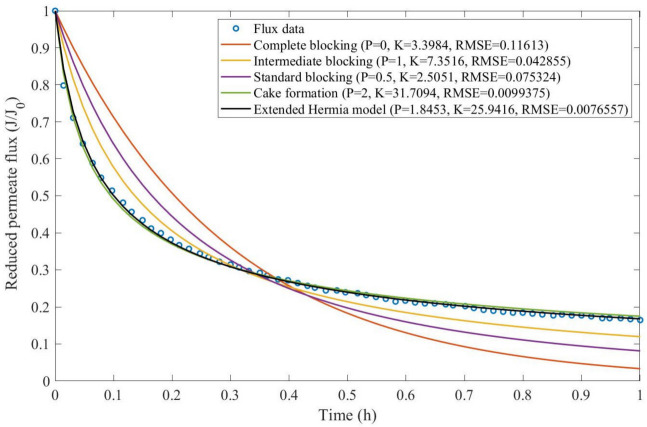
Model fitting for MIEX 12 mL/L + HPO ultrafiltration considering the four original pore-blocking mechanisms and the extended Hermia model (14).

**Figure A8:**
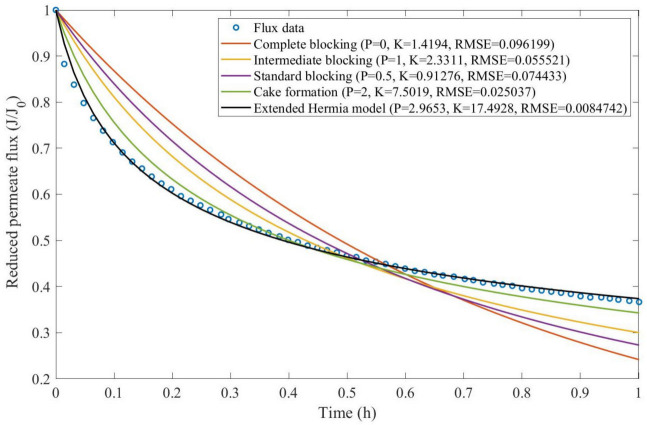
Model fitting for MIEX 12 mL/L + Coagulant 40 mg/L + HPO ultrafiltration considering the four original pore-blocking mechanisms and the extended Hermia model (14).

In this example, we isolated the data obtained from ultrafiltration for both HPI and HPO membranes with and without the addition of coagulant 140 mg/L and MIEX^®^ 12 mL/L. We performed the model fitting for all four pore-blocking mechanisms and the extended Hermia model by minimizing the root mean square error (RMSE). These regressions can be found in Appendix D (Figure A1, Figure A2, Figure A3, Figure A4, Figure A5, Figure A6, Figure A7 and Figure A8), and Table 2 and Table 3.

Based on the regressions obtained, we noticed that the extended Hermia model has a better performance when comparing the four blocking mechanisms (RMSE ≤ 0.01), followed by the cake formation mechanism. Although the EHM provides better estimates for flux, cases 1, 2, 3, 4, 5, 6, and 8 have P>2, which can be physically interpreted as a new blocking mechanism.

For cases 5 and 7 we obtained values of P that are relatively close to the cake formation mechanism, which implies that this type of blocking can happen to a certain degree. As an example, case 7 shows that 2≥P≥1; therefore, we can physically interpret this as a mixture of both cake formation and intermediate blocking. As for cases 3, 5, and 8, the same principle can be applied; therefore, these cases indicate a mixture of cake formation and a 3rd-degree blocking mechanism. Comparing both HPI and HPO membranes with no additions, the EHM predicts that the HPI membrane has a half-life of 0.57 h; meanwhile, the HPO membrane has a half-life of only 0.04 h. This indicates that for this example, fouling greatly affects HPO membranes when compared to HPI membranes. We also noticed that the addition of coagulant and MIEX^®^ increased the half-life for both HPI and HPO membranes.

This effect can be explained by the changes in the pore-blocking mechanism, since the values of P change with the addition of coagulant and MIEX^®^. With no additives, the mechanism tends toward cake formation (P=2.76 for HPI and P=2.23 for HPO), but the addition of coagulant shifts to a 4th-degree blocking mechanism for HPI and a 3rd-degree blocking mechanism for HPO (P=4.43 for HPI and P=3.04 for HPO). The addition of MIEX^®^ changes the blocking mechanisms slightly (P=2.88 for HPI and P=1.85 for HPO). As a result, we can infer that the most significant change to the pretreatment is the addition of the coagulant, which increases EMH half-life considerably by changing the pore-blocking mechanism. Therefore, given the results presented in Table 2, both additives used with the HPI membrane result in a considerable increase in EMH half-life, which indicates that this is a better solution for the fouling reduction in Example 1.

**Example** **2.** *Model fitting for microfiltration with ceramic membranes used in corn syrup clarification.*

**Figure A9:**
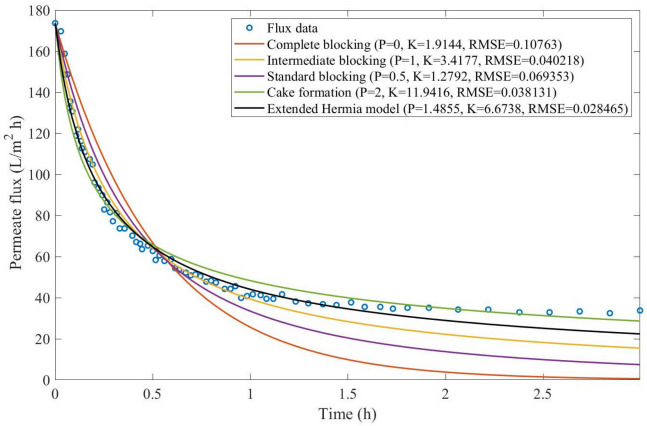
Model fitting for corn syrup microfiltration with CM08 at 0.5 m/s and 50 kPa considering the four original pore-blocking mechanisms and the extended Hermia model (2).

**Figure A10:**
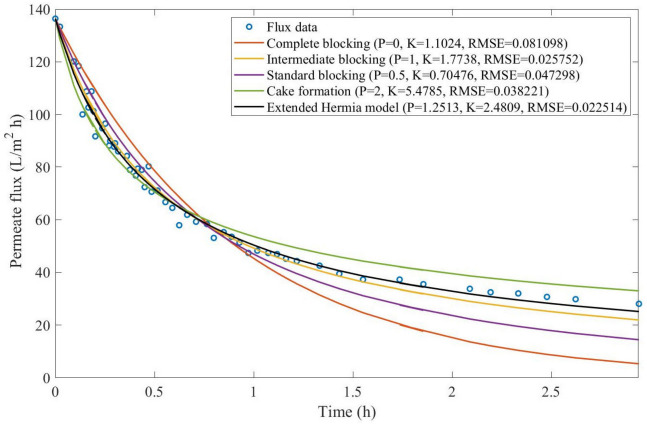
Model fitting for corn syrup microfiltration with CM05 at 0.5 m/s and 50 kPa considering the four original pore-blocking mechanisms and the extended Hermia model (2).

**Figure A11:**
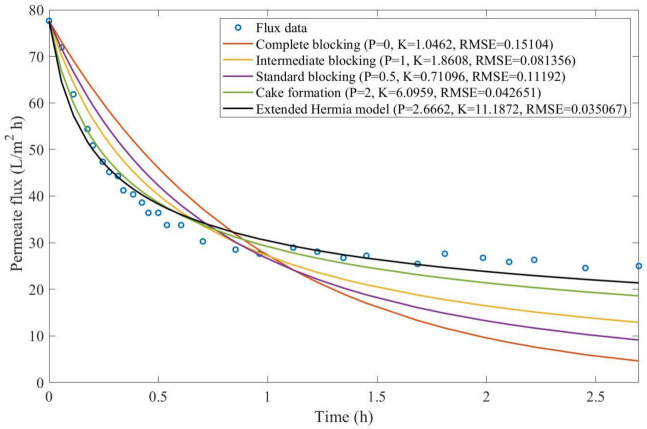
Model fitting for corn syrup microfiltration with CM01 at 0.5 m/s and 50 kPa considering the four original pore-blocking mechanisms and the extended Hermia model (2).

**Figure A12:**
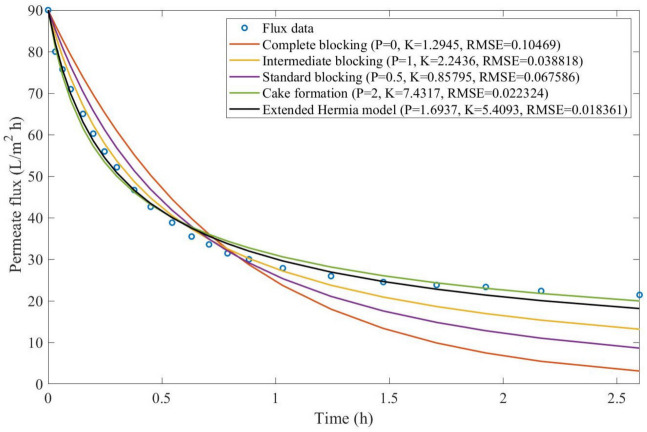
Model fitting for corn syrup microfiltration with CM05 at 2.31 m/s and 37.9 kPa considering the four original pore-blocking mechanisms and the extended Hermia model (2).

**Figure A13:**
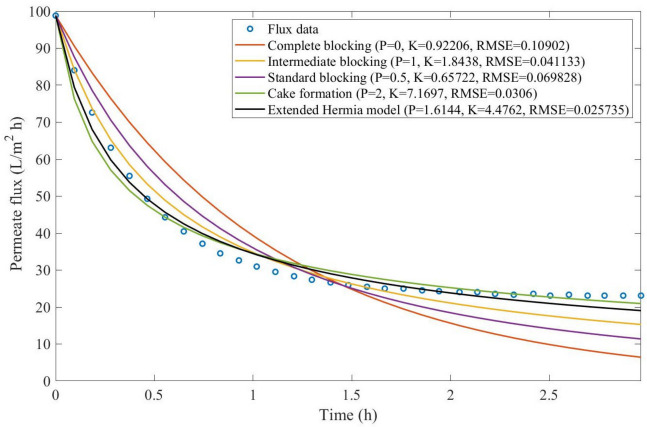
Model fitting for corn syrup microfiltration with CM05 at 2.31 m/s and 51.71 kPa considering the four original pore-blocking mechanisms and the extended Hermia model (2).

**Figure A14:**
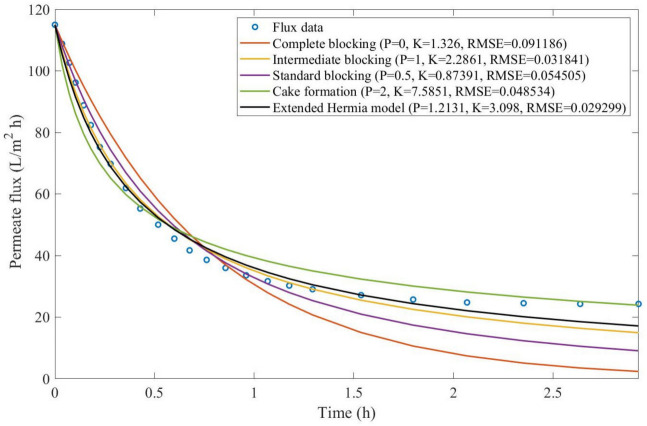
Model fitting for corn syrup microfiltration with CM05 at 2.31 m/s and 103.42 kPa considering the four original pore-blocking mechanisms and the extended Hermia model (2).

In a paper by Almandoz and coauthors, three different ceramic membranes (CM08, CM05, and CM01) were evaluated at different CFVs and TMPs for the removal of undesired oil, protein, and other non-starch components. The main difference between the ceramic membranes is their structure, mainly represented by properties such as mean pore radius obtained through volume mercury penetration ($r_{p}$), hydraulic permeability ($L_{h}$), and porosity (ε). Microfiltration was carried out at 0.5 m/s and 50 kPa for all three membranes, and CM05 was chosen for the following experiments due to better performance, including lower turbidity, lower concentrations of insoluble residues, and total proteins [33]. We recovered the data obtained throughout the experiments with CM08, CM05, and CM01 and performed the model fitting for all four pore-blocking mechanisms and the EHM. We also isolated the data for different TMP conditions for microfiltration with CM05. These results can be found in Appendix D (Figure A9, Figure A10, Figure A11, Figure A12, Figure A13 and Figure A14) and Table 4, Table 5, Table 6 and Table 7.

According to Table 4 and Table 5, the EHM performed better than the four classic pore-blocking mechanisms (RMSE ≤ 0.035), followed by cake formation (RMSE ≤ 0.042). We also noticed that the pore-blocking mechanism varies from membrane to membrane in the present example. Both CM08 and CM05 have a 1st-degree pore-blocking mechanism (between intermediate blocking and cake formation), while CM01 has a 2nd-degree blocking (between cake formation and a possible new type of pore-blocking). Meanwhile, the EMH half-life calculated for CM05 reveals that fouling does not affect this membrane as much as it does CM08 and CM01; therefore, this membrane was chosen by Almandoz and coauthors for later tests [2]. We consolidated the data from these later tests and performed the same analysis. The regression results are presented in Table 6 and Table 7.

Taking into consideration Table 6 and Table 7, the best-performing models are EHM, cake formation, and intermediate blocking. At times, intermediate blocking performs better than cake formation, yet the EHM still performs better than both. It is also interesting to point out that Figures have EHM with 2≥P≥1, indicating that a mixed blocking mechanism between intermediate blocking and cake formation can happen simultaneously. In this case, it seems that an increase in TMP causes a slight shift in the most prevalent blocking mechanism from cake formation to intermediate blocking, since P goes from 1.69 to 1.21. We noticed that the middle ground between cake formation and intermediate blocking slightly increases the EHM half-life, which is the desired outcome when optimizing the filtration conditions.

**Example** **3.** *Model fitting for cross-flow hollow-fiber ultrafiltration of oily effluent from a railway workshop.*

**Figure A15:**
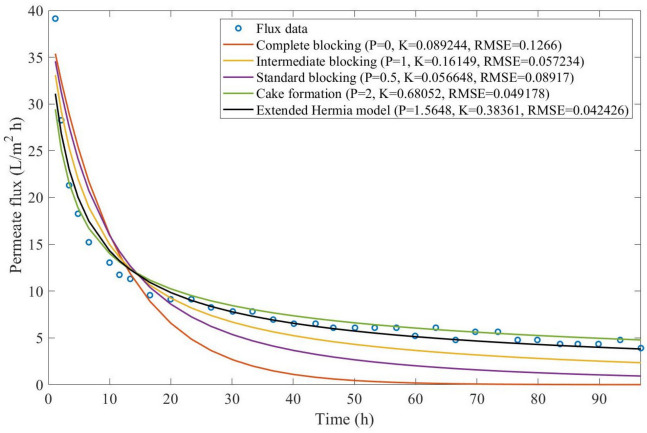
Model fitting for oily effluent ultrafiltration with CFR of 14 L/min and 21 kPa considering the four original pore-blocking mechanisms and the extended Hermia model (17).

**Figure A16:**
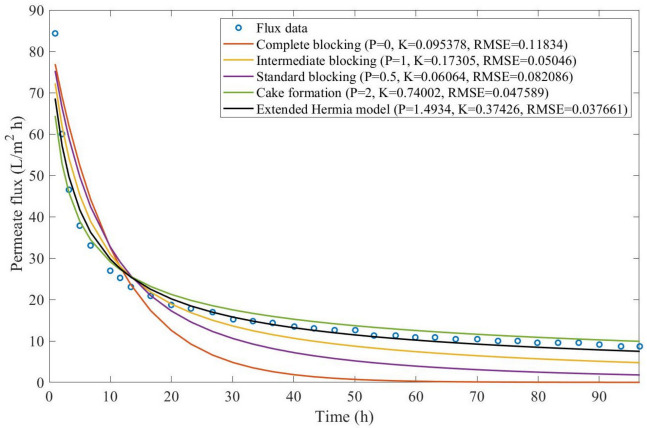
Model fitting for oily effluent ultrafiltration with CFR of 14 L/min and 35 kPa considering the four original pore-blocking mechanisms and the extended Hermia model (17).

**Figure A17:**
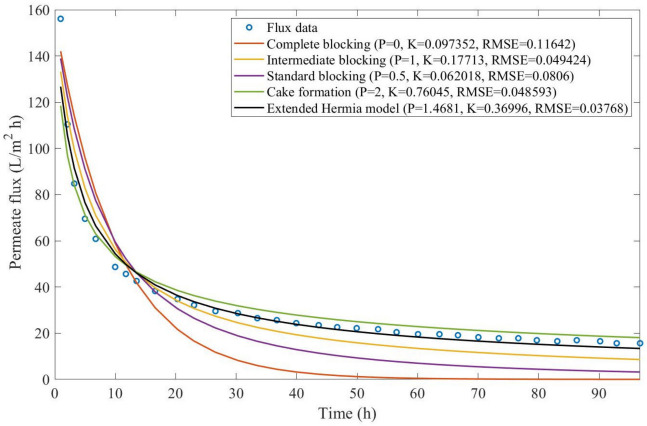
Model fitting for oily effluent ultrafiltration with CFR of 14 L/min and 104 kPa considering the four original pore-blocking mechanisms and the extended Hermia model (17).

**Figure A18:**
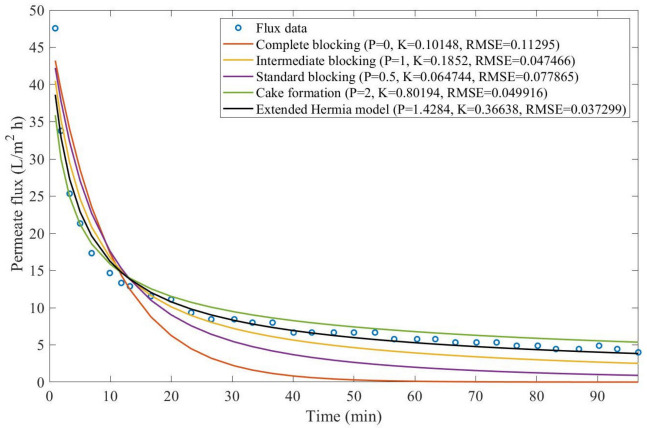
Model fitting for oily effluent ultrafiltration with CFR of 28 L/min and 21 kPa considering the four original pore-blocking mechanisms and the extended Hermia model (17).

**Figure A19:**
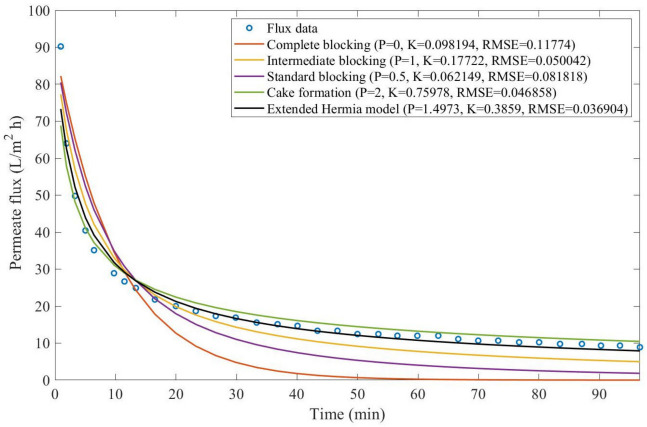
Model fitting for oily effluent ultrafiltration with CFR of 28 L/min and 35 kPa considering the four original pore-blocking mechanisms and the extended Hermia model (17).

**Figure A20:**
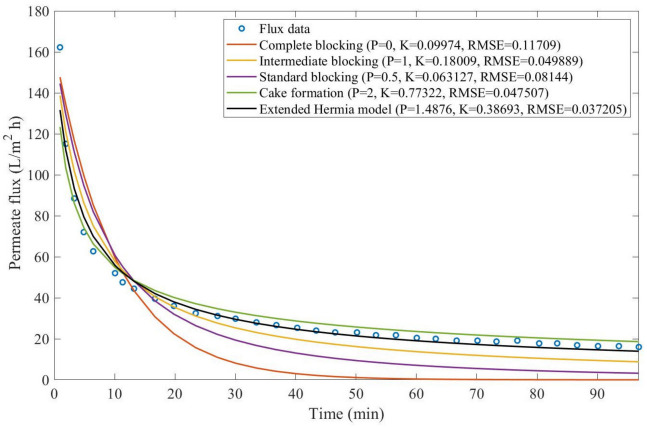
Model fitting for oily effluent ultrafiltration with CFR of 28 L/min and 104 kPa considering the four original pore-blocking mechanisms and the extended Hermia model (17).

**Figure A21:**
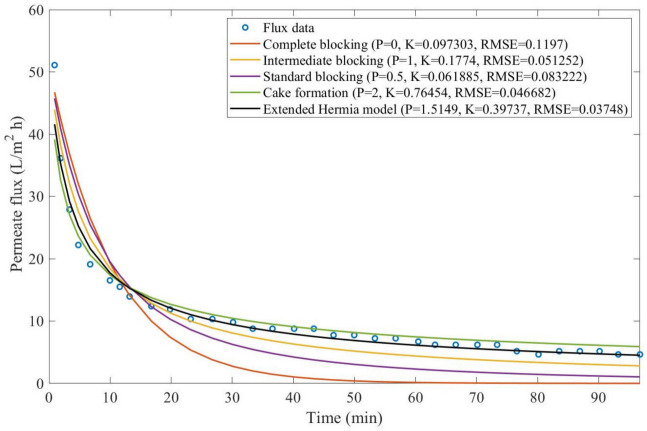
Model fitting for oily effluent ultrafiltration with CFR of 40 L/min and 21 kPa considering the four original pore-blocking mechanisms and the extended Hermia model (17).

**Figure A22:**
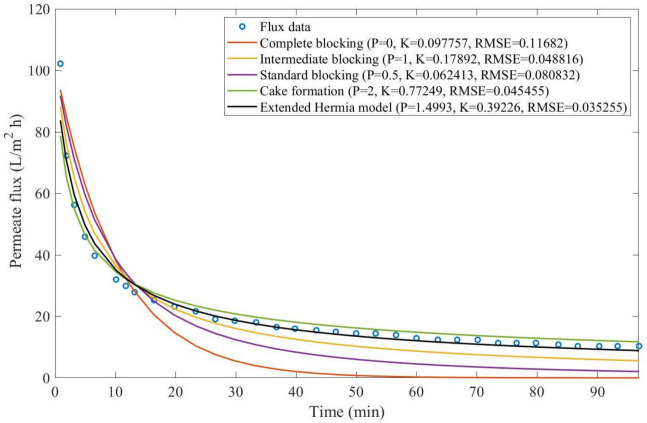
Model fitting for oily effluent ultrafiltration with CFR of 40 L/min and 35 kPa considering the four original pore-blocking mechanisms and the extended Hermia model (17).

**Figure A23:**
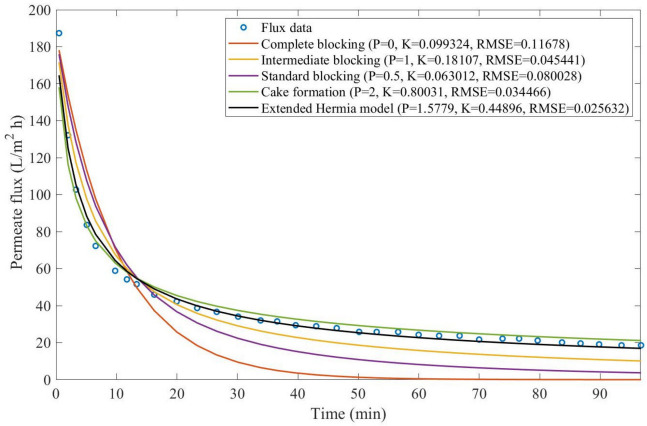
Model fitting for oily effluent ultrafiltration with CFR of 40 L/min and 104 kPa considering the four original pore-blocking mechanisms and the extended Hermia model (17).

In a paper by Kurada et al. [4], oily effluent containing dust, grease, and oil was treated by a sand bed followed by a cross-flow ultrafiltration hollow fiber membrane. Their experimental work involved changing both TMP (21–104 kPa) and CFR (14–40 L/min) and evaluating the aftermath, such as flux reduction and cake layer thickness. We extracted the data obtained by Kurada and Tanmay and applied the same techniques as presented in Examples 1 and 2. The regression results and EHM parameters can be found in Appendix D (Figure A15, Figure A16, Figure A17, Figure A18, Figure A19, Figure A20, Figure A21, Figure A22 and Figure A23) and Table 8 and Table 9.

Based on the regressions presented in Table 8 and Table 9, we observed very similar behavior to Example 2, in which the best performance was credited to the EHM (RMSE ≤ 0.042). Cake formation and intermediate blocking also performed well (RMSE ≤ 0.049 and ≤0.057 respectively). In Example 2, we pointed out that an increase in TMP changes the most prevalent pore-blocking mechanism from cake formation into intermediate blocking, because P had its value decreased. The same effect is also present here but only for a CFR of 14 L/min. For CFRs of 28 and 40 L/min, is seems that P behaves differently, increasing or decreasing with TMP. Changes in CFR while maintaining TMP constant also seems to have the same effect. Therefore, for the present example, it seems that significant changes in TMP and CFR do not change the pore-blocking mechanism considerably. We also noticed that decreasing both TMP and CFR leads to an increase in EHM half-life since, in this case, P tends toward cake formation, and lower values of TMP and CFR prevent the cake layer thickness from increasing as rapidly.

**Figure 3:**
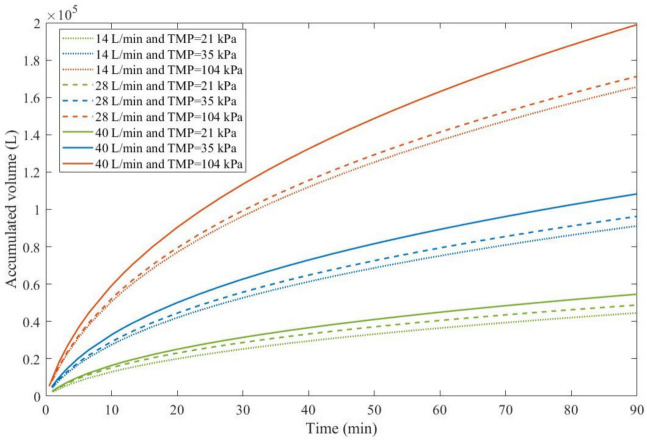
The accumulated volume calculated through the EHM for oily effluent ultrafiltration with CFR of 14–40 L/min and 21–104 kPa, assuming $A=1.00\;m^{2}$.

As a consequence, rather than looking at which experimental conditions lead to the most advantageous pore-blocking mechanism, we have to analyze which conditions result in higher values of $j_{0}$ and how this affects the accumulated permeate volume given in Equation (48) (Figure 3).

We color-coded Figure 3 for a better understanding. TMP is represented in different colors: green for 21 kPa, blue for 35 kPa, and red for 104 kPa. Full lines represent 40 L/min, discontinued lines represent 28 L/min, and dotted lines represent 14 L/min. Through Figure 3, we notice that an increase in TMP causes a general increase in the accumulated volume for all CFRs. For all TMPs we observed that an increase in CFR also causes an increase in the accumulated volume. Therefore, in Example 3, higher TMPs and CFRs are advantageous. It is important to point out that this effect is only possible because changes in both TMP and CFR do not change the blocking mechanism greatly, as shown in Table 8. For instance, in Example 4, we demonstrate that an increase in crossflow velocity can either increase or decrease the accumulated volume, depending on the blocking mechanism.

**Example** **4.** *Model fitting for ultrafiltration of alkali/surfactant/polymer flooding wastewater.*

In an experimental work by Ren et al., ultrafiltration was used to treat Alkali/surfactant/polymer (ASP) flooding wastewater, a commonly produced effluent in enhanced oil extraction processes that needs to be properly treated before reuse due to the potential threat of formation damage. In this study, the operating parameters were modified to research their effects on membrane fouling, which aimed to optimize the filtration conditions to minimize the effect of flux reduction. These parameters included TMP (2.12–2.79 bar) and CFV (0.75–3.00 m/s), with the ideal conditions being a TMP of 2.12 bar and CFV of 3.00 m/s [34].

**Figure A24:**
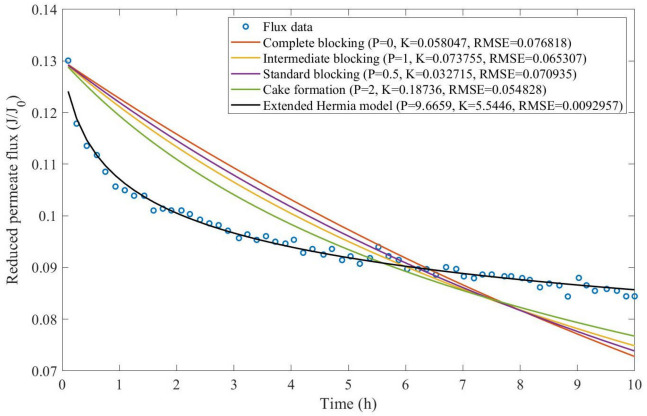
Model fitting for ultrafiltration of flooding wastewater at 2.12 bar and cross-flow velocity of 2.5 m/s considering the four original pore blocking mechanisms and the extended Hermia model (25).

**Figure A25:**
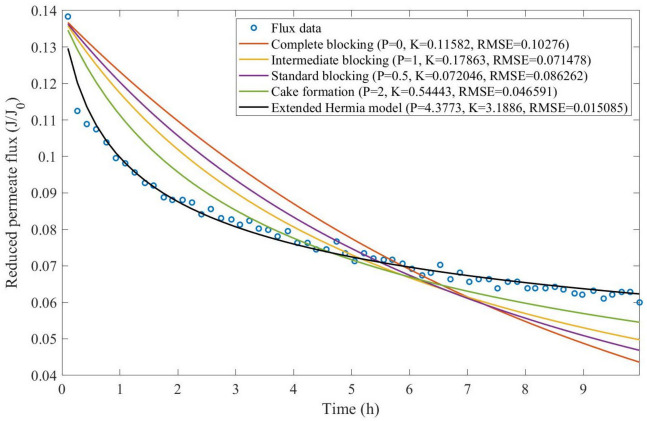
Model fitting for ultrafiltration of flooding wastewater at 2.79 bar and cross-flow velocity of 2.5 m/s considering the four original pore blocking mechanisms and the extended Hermia model (25).

**Figure A26:**
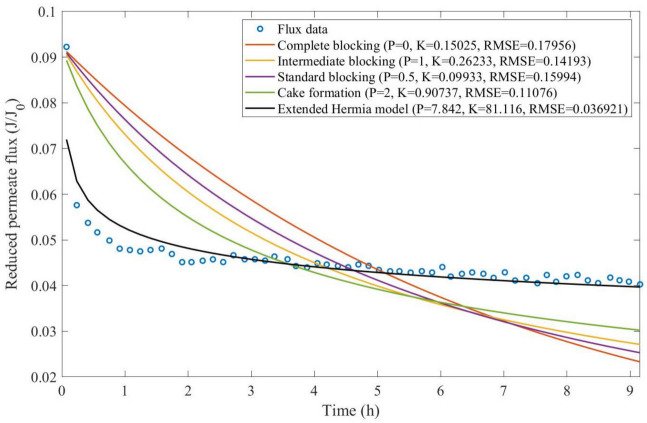
Model fitting for ultrafiltration of flooding wastewater at 2.20 bar and cross-flow velocity of 0.75 m/s considering the four original pore blocking mechanisms and the extended Hermia model (25).

**Figure A27:**
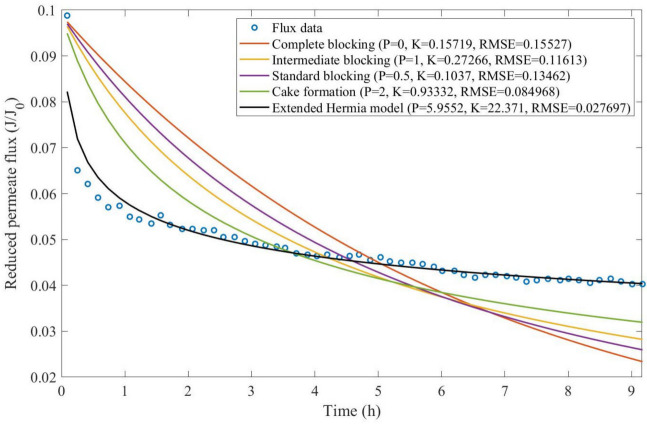
Model fitting for ultrafiltration of flooding wastewater at 2.20 bar and cross-flow velocity of 1.50 m/s considering the four original pore blocking mechanisms and the extended Hermia model (25).

**Figure A28:**
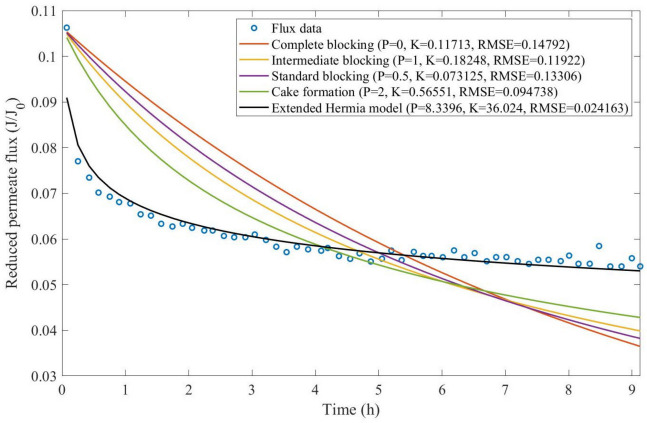
Model fitting for ultrafiltration of flooding wastewater at 2.20 bar and cross-flow velocity of 2.25 m/s considering the four original pore blocking mechanisms and the extended Hermia model (25).

**Figure A29:**
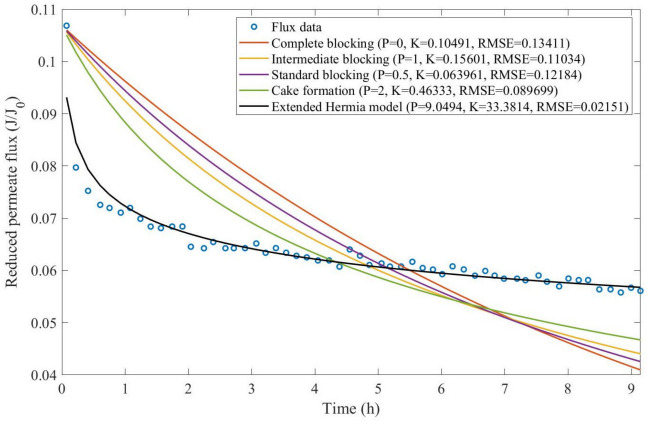
Model fitting for ultrafiltration of flooding wastewater at 2.20 bar and cross-flow velocity of 3.00 m/s considering the four original pore blocking mechanisms and the extended Hermia model (25).

We recovered the flux data obtained by Ren et al. and performed the model fitting for all four classic pore-blocking mechanisms and the EHM. These results are presented in Appendix D (Figure A24, Figure A25, Figure A26, Figure A27, Figure A28 and Figure A29) and Table 10, Table 11, Table 12 and Table 13.

According to Table 10 and Table 11, across all the model fittings, the EHM presents the best data fit (RMSE ≤ 0.015), as the other pore-blocking mechanisms do not seem to fit the data accurately. We noticed that an increase in TMP causes a decrease in the value of P, changing the pore blocking mechanism from a 9th degree to a 4th degree. The present example was included to demonstrate that even though the EHM fits the data well, it is important to exercise caution. The EHM half-life, when calculated using Equation (46), yields results that are not physically accurate. Since the data recovered from Ren et al. does not include $\frac{j}{j_{0}}>0.14$ in Figure A24, Figure A25, Figure A26, Figure A27, Figure A28 and Figure A29, the values obtained for P and K do not represent values $\frac{j}{j_{0}}>0.14$. Therefore, the use of Equation (46) extrapolates the model for points that were not included in the regression, which results in EHM half-life values that are non-representative. The same regressions were performed on the experimental tests at 2.20 bar with varying CFV (0.75–3.00 m/s) (Table 12 and Table 13).

**Figure 4:**
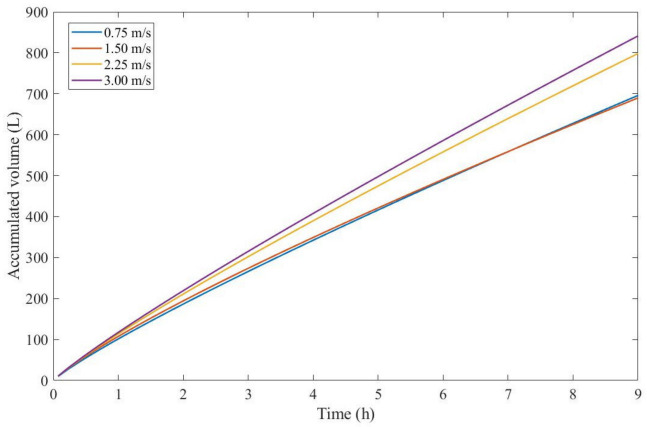
The accumulated volume calculated through the EHM for ultrafiltration of flooding wastewater at 2.20 bar and cross-flow velocities of 0.75–3.00 m/s, assuming $j_{0}=100\;L/m^{2}h$ and $A=1.567\;m^{2}$.

Taking into account Table 12 and Table 13, we observed that the EHM had a better performance (RMSE ≤ 0.037) when compared to the four original pore-blocking mechanisms. For the present example, it seems that changes in CFV affect P greatly, changing between 5th-degree and 9th-degree mechanisms. Once again, the EHM half-life is not physically representative because it is extrapolating the model, such as in Table 10; therefore, if this happens in the following Examples (Examples 5 and 6), the EHM half-life will be referred to as non-applicable (N/A). One alternative to better rank the filtration conditions to optimize the process is to use Equation (48). By calculating the accumulated volume of permeate, it is possible to obtain a function of P and K, making it possible to rank the filtration conditions through accumulated volume maximization. Figure 4 presents the accumulated volume calculated using Equation (48) assuming $j_{0}=100\;L/m^{2}h$ and $A=1.567\;m^{2}$ for all CFVs.

Through Figure 4, we calculated that an accumulated volume for a CFV of 3.00 m/s yields better results; therefore a 9th-degree pore-blocking mechanism is favorable in this context. Due to the non-linear nature of Equation (48), a higher CFV will not always provide higher values for the accumulated volume, as shown in Figure 4 for CFVs of 0.75 and 1.50 m/s. At one point, both of their curves meet, which means that, at times, fouling will affect the membrane to such a degree that lower CFVs would yield a higher permeate production.

**Example** **5.** *Model fitting for ultrafiltration of bovine serum albumin solutions.*

Aiming to decrease the effects of fouling in iron oxide ultrafiltration membranes, Storms and collaborators coated these ceramic membranes with poly(sulfobetaine methacrylate) (polySBMA), a superhydrophilic zwitterionic polymer, and investigated whether this modification was helpful towards flux reduction. Albumin solutions were filtered at a TMP of 103.421 kPa in three fouling stages for both uncoated and coated membranes, such that washings were performed between stages [10].

**Figure A30:**
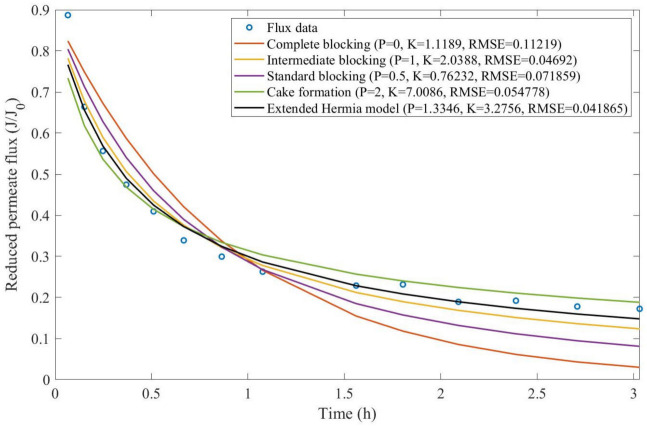
Model fitting for the first fouling stage of ultrafiltration of bovine serum albumin solution at 103.421 kPa considering the four original pore-blocking mechanisms and the extended Hermia model (Uncoated membrane) (29).

**Figure A31:**
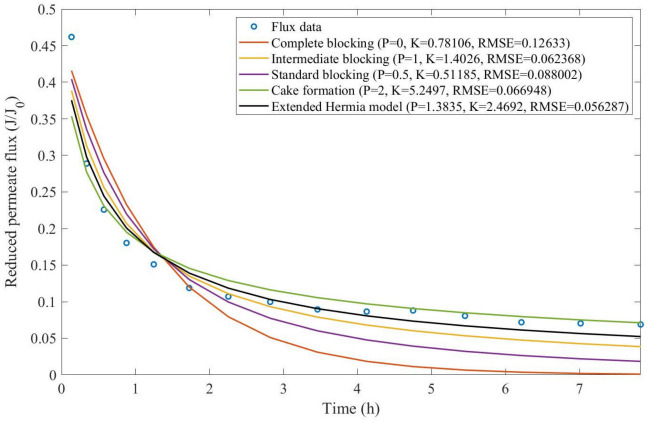
Model fitting for the second fouling stage of ultrafiltration of bovine serum albumin solution at 103.421 kPa considering the four original pore-blocking mechanisms and the extended Hermia model (Uncoated membrane) (29).

**Figure A32:**
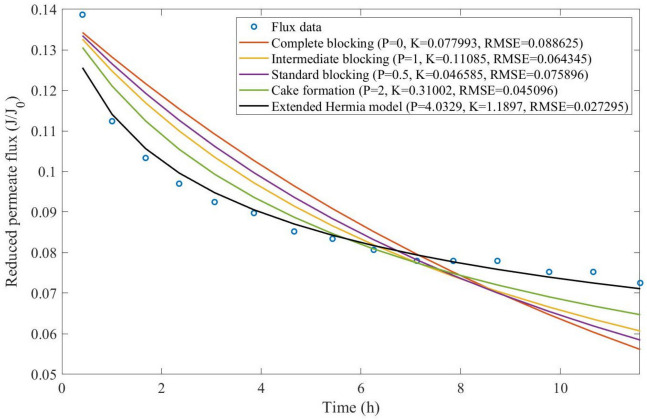
Model fitting for the third fouling stage of ultrafiltration of bovine serum albumin solution at 103.421 kPa considering the four original pore-blocking mechanisms and the extended Hermia model (Uncoated membrane) (29).

**Figure A33:**
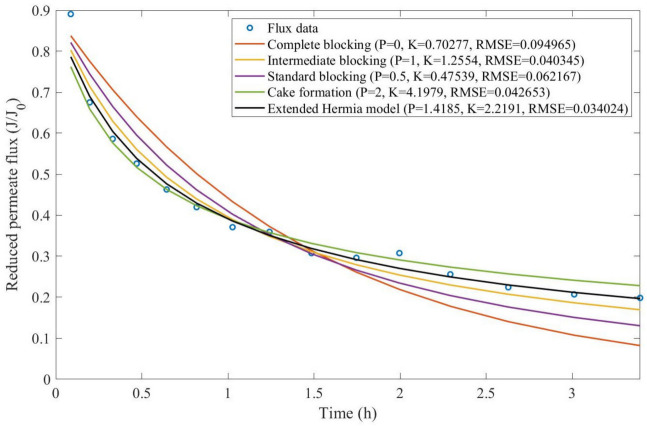
Model fitting for the first fouling stage of ultrafiltration of bovine serum albumin solution at 103.421 kPa considering the four original pore-blocking mechanisms and the extended Hermia model (Coated membrane) (29).

**Figure A34:**
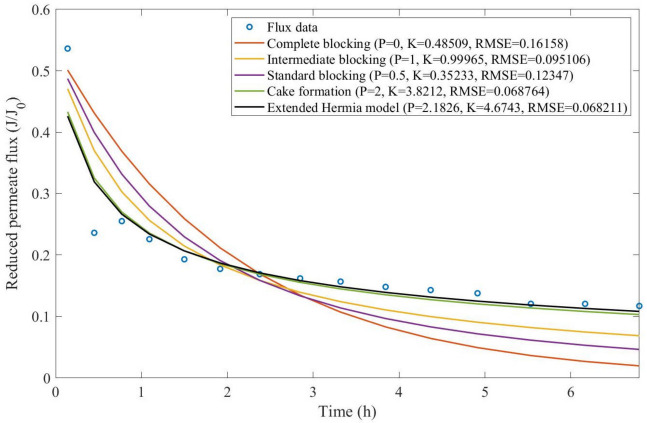
Model fitting for the second fouling stage of ultrafiltration of bovine serum albumin solution at 103.421 kPa considering the four original pore-blocking mechanisms and the extended Hermia model (Coated membrane) (29).

**Figure A35:**
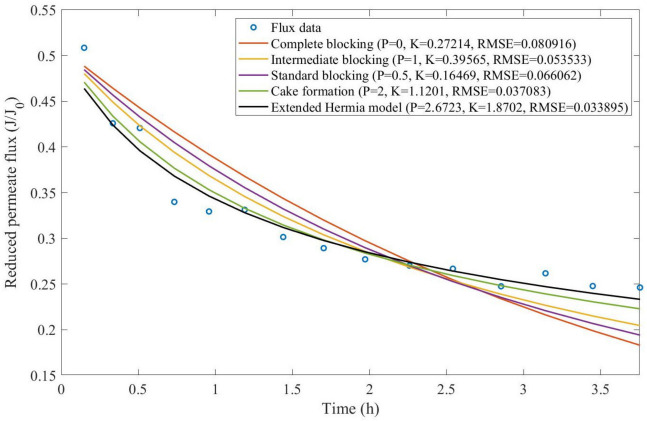
Model fitting for the third fouling stage of ultrafiltration of bovine serum albumin solution at 103.421 kPa considering the four original pore-blocking mechanisms and the extended Hermia model (Coated membrane) (29).

We recovered the experimental data obtained for the three fouling stages for both uncoated and coated membranes. The same model fitting performed in Examples 1–4 was also applied to the present example. The regressions obtained are presented in Appendix D (Figure A30, Figure A31, Figure A32, Figure A33, Figure A34 and Figure A35) and Table 14 and Table 15.

By the results presented in Table 14 and Table 15, we demonstrated that the EHM performs better than the original four pore-blocking mechanisms, since it has an RMSE ≤ 0.068. According to the values obtained for P, the addition of polybag changes the pore-blocking mechanism for the first fouling stage, which starts with a prevalent mechanism of intermediate blocking and changes slightly to cake formation, since P goes from 1.33 to 1.42. This change is further supported by the second fouling stage, in which P=1.38 for the uncoated membrane and 2.18 for the coated membrane. The third fouling stage shows that the uncoated membrane encounters a big shift in the pore-blocking mechanism, going from a mainly intermediate blocking (1st degree) to a 4th degree. In contrast, the third fouling stage for the coated membrane remains mainly as a cake formation mechanism (2nd degree).

We also observed an increase in EHM half-life in the first and second fouling stages, which indicated that the addition of polySBMA does mitigate fouling to a certain degree. It is also interesting to point out that the polySBMA coating seems to cause a cake formation mechanism, which, in this case, is advantageous since it increases the EHM half-life and the amount of permeate obtained per filtration batch.

**Example** **6.** *Model fitting for ultrafiltration of nanoparticles from polishing wastewater.*

In a series of ultrafiltration experiments at a laboratory scale conducted by Ohanessian et al., chemical mechanical polishing wastewater filtration was carried out to optimize and validate fouling models. Two types of experiments were performed: dead-end filtration at a TMP of 0.4 bar and crossflow filtration at a TMP of 0.3 bar. Different concentrations were evaluated for both: 97, 251, and 657 mgNPs/L (milligrams of nanoparticles per liter) for dead-end filtration and 332, 572, and 2600 mgNPs/L for crossflow filtration [35].

**Figure A36:**
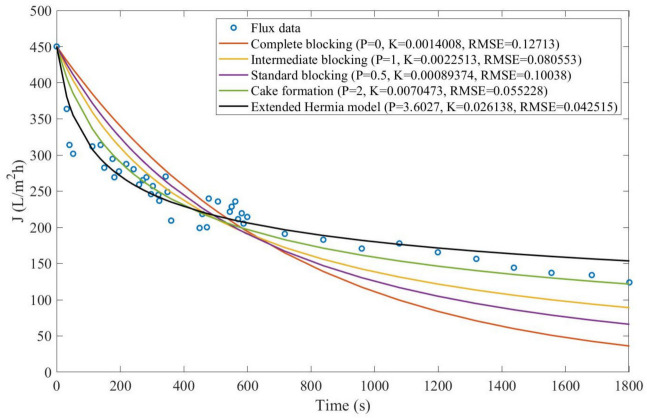
Model fitting for dead-end ultrafiltration of nanoparticles (97 mgNPs/L) from polishing wastewater at 0.4 bar considering the four original pore-blocking mechanisms and the extended Hermia model (22).

**Figure A37:**
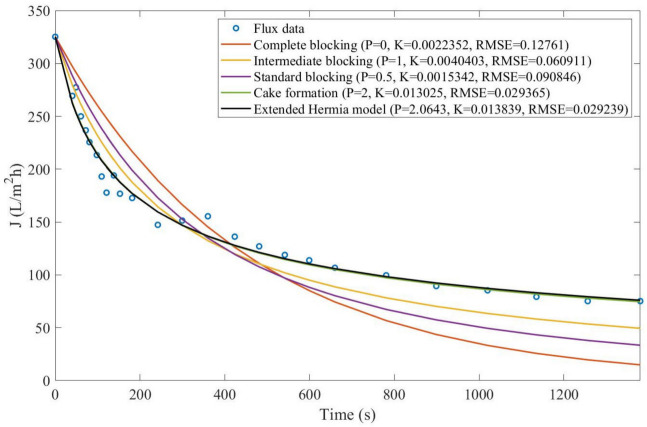
Model fitting for dead-end ultrafiltration of nanoparticles (251 mgNPs/L) from polishing wastewater at 0.4 bar considering the four original pore-blocking mechanisms and the extended Hermia model (22).

**Figure A38:**
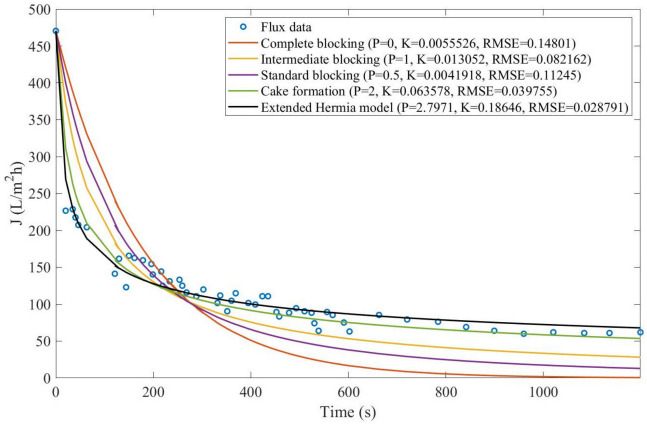
Model fitting for dead-end ultrafiltration of nanoparticles (657 mgNPs/L) from polishing wastewater at 0.4 bar considering the four original pore-blocking mechanisms and the extended Hermia model (22).

We recovered the data from the dead-end filtration experiments and performed the model fitting for all original pore-blocking mechanisms, as well as the EHM. The regression results are presented in Appendix D (Figure A36, Figure A37 and Figure A38) and Table 16 and Table 17.

According to Table 16 and Table 17, throughout the experiment, the EHM consistently performed better than the original pore-blocking mechanisms (RMSE ≤ 0.042), followed closely by the cake formation mechanism (RMSE ≤ 0.055). We also noticed that an increase in the concentration of nanoparticles non-linearly changes the pore-blocking mechanism, starting at a 3rd degree and moving to a mostly cake formation mechanism (2nd degree). In contrast, a further increase in nanoparticle concentration does the opposite, changing from mostly cake formation to a mixed pore-blocking mechanism of cake formation and 3rd-degree blocking.

**Figure 5:**
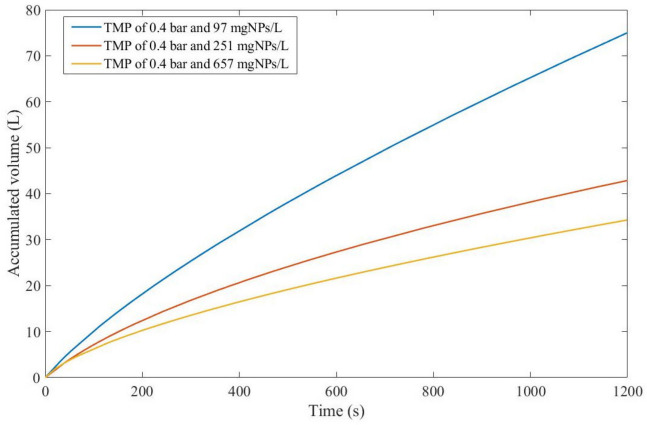
The accumulated volume calculated through the EHM for dead-end ultrafiltration of nanoparticles from polishing wastewater at 0.4 bar, assuming $A=1.00{\;m}^{2}$.

Taking into account the EHM half-life, it seems that an increase in nanoparticle concentration is directly correlated with a decrease in half-life, which indicates that fouling has a greater effect at higher concentrations. It is important to point out that the flux data presented in Figure A36, Figure A37 and Figure A38 have different values of $j_{0}$ for each concentration; therefore, to better classify which pore-blocking mechanism is the most advantageous in Example 6, we used Equation (48) and the regression results to calculate the accumulated permeate volume (Figure 5), assuming $A=1\;m^{2}$.

Even though $j_{0}$ for 657 mgNPs/L is greater than $j_{0}$ for 97 mgNPs/L, Figure 5 shows that the accumulated volume obtained through a concentration of 97 mgNPs/L is far greater than for higher concentrations. This implies that the effects of fouling are more pronounced for higher concentrations, as demonstrated through EHM half-life. Therefore, we can conclude that, in Example 6, a 3rd-degree pore-blocking mechanism at lower concentrations for dead-end ultrafiltration of nanoparticles at 0.4 bar is advantageous.

## 4. Discussion

### 4.1. How the Blocking Mechanism Changes with Membrane Types and Pre-Treatments

In the same filtration conditions, different membrane types also have different blocking mechanisms, such as shown in Example 1, where HPI and HPO membranes behaved differently. This behavior was recurrent in Example 1, where changes to both the foulant and the fluid caused changes in fouling for both HPI and HPO. Other membrane properties also influence the blocking mechanism, such as shown in Example 2, where the same type of ceramic membrane performed differently due to differences in mean pore radius obtained through volume mercury penetration ($r_{p}$), hydraulic permeability ($L_{h}$), and porosity (ε). Membrane usage also plays a big role, since multiple fouling stages also change the blocking mechanism. The use of coatings also has effects on the pore-blocking mechanism, such as shown in Example 5.

A common factor in Examples 1, 2, and 5 is the changes in the interactions between the foulant and the membrane itself. HPI and HPO have different van der Walls interactions with both the foulant and the fluid, the use of coagulants and additives changes the size distribution of particles, different mean pore radius influences the membrane’s selectivity, and changes to the membrane’s surface interfere also change how fouling layers behave when in contact with the membrane. Therefore, given all possible changes that can be made to an experimental setup, the influence of these changes in the pore-blocking mechanism is very situation-specific.

For instance, in Example 1, the experimental conditions that maximized EHM half-life were the use of both additives with the HPI membrane, changing the pore-blocking mechanism from a mixture of cake formation and a 3rd-degree mechanism to mainly 3rd degree. In contrast, in Example 5, the coated membrane maximized EHM half-life by changing the pore-blocking mechanism from a mixture of intermediate blocking and cake formation to mainly cake formation.

### 4.2. How the Blocking Mechanism Changes with TMP, CFR, CFV, and Matter Concentration

The effects of TMP in the pore-blocking mechanism seem to vary in intensity, as shown in Examples 2–4. In Example 2, an increase in TMP for CM05 causes a decrease in the value of P, changing the blocking mechanism from mainly cake formation to mainly intermediate blocking. In Example 3 at a CFR of 14 L/min, an increase in TMP leads to a slight decrease in P; meanwhile, at CFRs of 28 and 40 L/min, P seems to slightly increase. In contrast, in Example 4, a smaller increase in TMP leads to P decreasing by almost half. In Examples 2 and 4, increasing TMP seems to decrease P. The changes in TMP applied in Example 3 do not cause significant changes in P; therefore, we can suggest that P is inversely proportional to TMP; however, further use of the EHM is necessary to confirm this statement.

Changes in CFR, and consequently CFV, seem to vary with TMP. In Example 3, at a TMP of 21 kPa, an increase in CFR leads to a decrease in P. This behavior changes for TMPs of 35 and 104 kPa, where an increase in CFR leads to a decrease in P. The same type of mixed behavior was identified in Example 4, where an increase in CFV from 0.75 to 1.50 m/s leads to a decrease in P, yet a further increase from 1.50 to 3.00 m/s causes an increase.

Similar non-linear effects can be found in changes in concentration, such as in Example 6. An increase in nanoparticle concentration from 97 to 251 mgNPs/L decreases P, changing the blocking mechanism from a 3rd-degree to cake formation. Yet, further increase from 251 to 657 mgNPs/L increases P, changing the blocking mechanism from cake formation to a mixture of cake formation and a 3rd-degree blocking mechanism.

Taking into account Examples 2–4 and 6, we show that the same types of changes in the operating conditions of different filtration systems lead to vastly different fouling behavior and pore-blocking mechanisms. Therefore, the use of P as a tool to better understand fouling in membranes needs to be accompanied by auxiliary variables that indicate different performances, such as the EHM half-life, accumulated volume, matter concentration measurements, and so on.

### 4.3. Higher-Degree and Mixed Pore-Blocking Mechanisms

In Examples 1, 2, 4, 5, and 6, we found optimal values of P that were higher than 2nd-degree blocking (cake formation). In other words, there are values of P>2. Through its connection to n, there seem to be not only values of n between the original four blocking mechanisms (n=0,1,3/2,2) but also values where n<0. The standard physical interpretation is for these exact values, such that complete blocking is n=2, intermediate blocking is n=1, standard blocking is n=3/2, and cake formation n=0. It is possible to interpret the values in between (i.e., n=0.75) as a mixture of the pore-blocking mechanisms (i.e., cake formation and intermediate blocking). This interpretation is used in all examples. The physical interpretation for values of n<0 (or P>2) requires more experimental work to fully understand what these new possible pore-blocking mechanisms look like and how they contribute to membrane fouling as a whole.

### 4.4. Fouling Mitigation, Optimal Filtration Conditions, and Physically Representative Use of the EHM

Given Equations (46) and (48) from Theorems 1 and 3, both the EHM half-life and the accumulated volume increase with P and decreases with k; therefore, to increase the half-life of the membrane, it is possible to apply many strategies that increase the degree of the pore-blocking mechanism, such as the ones applied in Examples 1–6. It is important to point out that given the non-linearity of the conditions, such as shown in Section 4.2, optimizations should follow a systematic approach, perhaps given by experimental design tools and statistical analysis. We showed in Example 4 that the EHM can be used to predict interpolated values, yet extrapolations can lead to inconclusive results. Therefore, the representative use of the model depends on the data used for the model fitting. Throughout Examples 1–6, there were cases in which more than one pore-blocking mechanism could be used to explain fouling, given by the lower values obtained for RMSE. In contrast, the EHM fitting provided the best solution possible for P. Thus, since P is a parameter that takes into account the whole dataset, it can be interpreted as a measurement of the possible blocking mechanisms throughout the filtration process. Still, depending on the filtration conditions and the experimental setup, assigning a physical pore-blocking mechanism to the values of P obtained can be a challenge.

## 5. Conclusions

Theorems 1–3 were tested in Examples 1–6 to compare the performance of the EHM to the original Hermia model. The following results were obtained in this study:

- The Hermia model can be used for any real values of n and k for equal or approximate entrance and exit densities. The reduced permeate flux j is given by a power law dependent on the value of n for any real n≠2 and by an exponential function when n=2. The EHM performed consistently better than the four original pore-blocking mechanisms in all examples;
- The effects of membrane composition, solution nature, TMP, CFR, and CFV greatly impact the values of P and k; therefore, the fouling behavior is situation-specific, and P and k may vary differently with the same variable in different cases;
- The EHM can be used to interpolate data, but extrapolating data can lead to inconclusive numerical results;
- There seem to be not only values of n between the original four blocking mechanisms (n=0,1,3/2,2) but also values where n<0. It is possible to interpret the values in between (i.e., n=0.75) as a mixture of the pore-blocking mechanisms (i.e., cake formation and intermediate blocking). This interpretation is used in all Examples. As for values of n<0, more research needs to be done in this area to better understand the physical meaning behind this phenomenon.

## Figures and Tables

**Table 1:** Application examples of the Hermia model.

Filtration	TMP	Pore-Blocking Mechanism	Performance	Reference
Ultrafiltration of polyethylene glycol	0.1, 0.2, 0.3, and 0.4 MPa	CB	R^2^ between 0.621 and 0.913	[2]
		IB	R^2^ between 0.638 and 0.923	
		SB	R^2^ between 0.635 and 0.918	
		CF	R^2^ between 0.639 and 0.947	
Ultrafiltration of glycerin–water solutions	2 bar	CB	R^2^ between 0.695 and 0.861	[3]
		IB	R^2^ between 0.861 and 0.962	
		SB	R^2^ between 0.799 and 0.932	
		CF	R^2^ between 0.728 and 0.957	
Ultrafiltration for treatment of effluent from a railway workshop	21, 35, and 48 kPa	CB	R^2^ between 0.75 and 0.88	[4]
		IB	R^2^ between 0.88 and 0.92	
		SB	R^2^ between 0.83 and 0.91	
		CF	R^2^ between 0.97 and 0.98	
Ultrafiltration of effluent organic matter	0.03 MPa	CB	R^2^ between 0.695 and 0.832	[6]
		IB	R^2^ between 0.672 and 0.821	
		SB	R^2^ between 0.994 and 0.997	
		CF	R^2^ between 0.752 and 0.952	
Filtration of microalgae	8, 9, and 10 kPa	CB	R^2^ between 0.740 and 0.883	[5]
		IB	R^2^ between 0.819 and 0.899	
		SB	R^2^ between 0.782 and 0.888	
		CF	R^2^ between 0.874 and 0.921	
Nanofiltration of polycyclic aromatic hydrocarbons	4.5 bar	CB	R^2^ of 0.863 and 0.978	[7]
		IB	R^2^ of 0.936 and 0.988	
		SB	R^2^ of 0.947 and 0.991	
		CF	R^2^ of 0.908 and 0.957	

**Table 2:** EHM parameters for the regressions obtained in Example 1.

Case	Filtration Conditions	P	K (h^−1^)	EHM Half-Life (h)
1	HPI only	2.76	10.22	0.57
2	Coag. 140 mg/L + HPI	4.43	9.56	2.15
3	MIEX 12 mL/L	2.88	9.33	0.68
4	MIEX 12 mL/L + Coag. 40 mg/L + HPI	3.08	2.41	3.11
5	HPO only	2.23	99.20	0.04
6	Coag. 140 mg/L + HPO	3.04	24.82	0.29
7	MIEX 12 mL/L + HPO	1.85	25.94	0.10
8	MIEX 12 mL/L + Coag. 40 mg/L + HPO	2.97	17.49	0.39

**Table 3:** RMSE results obtained in Example 1.

Case	Filtration Conditions	CB	IB	SB	CF	EHM
1	HPI only	0.0795	0.0449	0.0612	0.0178	0.0048
2	Coag. 140 mg/L + HPI	0.0593	0.0429	0.0508	0.0287	0.0065
3	MIEX 12 mL/L	0.0725	0.0423	0.0566	0.0184	0.0054
4	MIEX 12 mL/L + Coag. 40 mg/L + HPI	0.025	0.0164	0.0206	0.0090	0.0046
5	HPO only	0.13320	0.0598	0.0936	0.0134	0.0101
6	Coag. 140 mg/L + HPO	0.1104	0.0634	0.0851	0.0287	0.0078
7	MIEX 12 mL/L + HPO	0.1161	0.0429	0.0753	0.0099	0.0077
8	MIEX 12 mL/L + Coag. 40 mg/L + HPO	0.0962	0.0555	0.0744	0.025	0.0085

**Table 4:** EHM parameters for the regressions obtained in Example 2 for CM08, CM05, and CM01.

Membrane	P	K (h^−1^)	EHM Half-Life (h)
CM08	1.49	6.67	0.27
CM05	1.25	2.48	0.56
CM01	2.67	11.19	0.48

**Table 5:** RMSE results obtained in Example 2 for CM08, CM05, and CM01.

Membrane	CB	IB	SB	CF	EHM
CM08	0.1076	0.0402	0.0694	0.0381	0.0285
CM05	0.0811	0.0258	0.0473	0.0382	0.0225
CM01	0.151	0.0814	0.1119	0.0427	0.0351

**Table 6:** EHM parameters for the regressions obtained in Example 2 for CM05 at different TMPs.

TMP	P	K (h^−1^)	EHM Half-Life (h)
103.42 kPa	1.21	3.08	0.43
51.71 kPa	1.61	4.48	0.46
37.9 kPa	1.69	5.41	0.41

**Table 7:** RMSE results obtained in Example 2 for CM05 at different TMPs.

TMP	CB	IB	SB	CF	EHM
103.42 kPa	0.0912	0.0318	0.0545	0.0485	0.0293
51.71 kPa	0.1090	0.0411	0.0698	0.0306	0.0257
37.9 kPa	0.1047	0.0388	0.0676	0.0223	0.0184

**Table 8:** EHM parameters for the regressions obtained in Example 3.

Filtration Conditions	P	K (min^−1^)	EHM Half-Life (min)
CFR 14 L/min 21 kPa	1.56	0.38	5.10
CFR 14 L/min 35 kPa	1.49	0.37	4.85
CFR 14 L/min 104 kPa	1.47	0.37	4.77
CFR 28 L/min 21 kPa	1.43	0.37	4.62
CFR 28 L/min 35 kPa	1.50	0.39	4.72
CFR 28 L/min 104 kPa	1.49	0.39	4.66
CFR 40 L/min 21 kPa	1.51	0.40	4.68
CFR 40 L/min 35 kPa	1.50	0.39	4.66
CFR 40 L/min 104 kPa	1.58	0.45	4.42

**Table 9:** RMSE results obtained in Example 3.

Filtration Conditions	CB	IB	SB	CF	EHM
CFR 14 L/min 21 kPa	0.1266	0.0572	0.0892	0.0492	0.0424
CFR 14 L/min 35 kPa	0.1183	0.0505	0.0821	0.0476	0.0377
CFR 14 L/min 104 kPa	0.1164	0.0494	0.0806	0.0486	0.0377
CFR 28 L/min 21 kPa	0.1129	0.0475	0.0779	0.0499	0.0373
CFR 28 L/min 35 kPa	0.1177	0.0500	0.0818	0.0469	0.0369
CFR 28 L/min 104 kPa	0.1171	0.0499	0.0814	0.0475	0.0372
CFR 40 L/min 21 kPa	0.1197	0.0513	0.0832	0.0467	0.0375
CFR 40 L/min 35 kPa	0.1168	0.0488	0.0808	0.0455	0.0353
CFR 40 L/min 104 kPa	0.1168	0.0454	0.0800	0.0345	0.0256

**Table 10:** EHM parameters for the regressions obtained in Example 4 at 2.5 m/s and different TMPs.

Filtration Conditions	P	K (h^−1^)	EHM Half-Life (h^−1^)
2.12 bar	9.67	5.54	146.33
2.79 bar	4.38	3.19	6.20

**Table 11:** RMSE results obtained in Example 4 at 2.5 m/s and different TMPs.

Filtration Conditions	CB	IB	SB	CF	EHM
2.12 bar	0.0768	0.0653	0.0709	0.0548	0.00930
2.79 bar	0.1028	0.0715	0.0863	0.0466	0.0151

**Table 12:** EHM parameters for the regressions obtained in Example 4 for different cross-flow velocities.

Filtration Conditions	P	K (h^−1^)	EHM Half-Life (h)
0.75 m/s	7.84	81.12	2.82
1.50 m/s	5.96	22.37	2.73
2.25 m/s	8.34	36.02	8.96
3.00 m/s	9.05	33.38	15.84

**Table 13:** RMSE results obtained in Example 4 for different cross-flow velocities.

Filtration Conditions	CB	IB	SB	CF	EHM
0.75 m/s	0.1796	0.1419	0.1599	0.1108	0.0369
1.50 m/s	0.1553	0.1161	0.1346	0.0850	0.0277
2.25 m/s	0.1479	0.1192	0.1331	0.0947	0.0242
3.00 m/s	0.1341	0.1103	0.1218	0.0897	0.0215

**Table 14:** EHM parameters for the regressions obtained in Example 5.

Filtration Conditions	P	K (h^−1^)	EHM Half-Life (h)
First fouling stage (Uncoated membrane)	1.33	3.28	0.46
Second fouling stage (Uncoated membrane)	1.38	2.47	0.65
Third fouling stage (Uncoated membrane)	4.03	1.19	N/A
First fouling stage (Coated membrane)	1.42	2.22	0.75
Second fouling stage (Coated membrane)	2.18	4.67	0.76
Third fouling stage (Coated membrane)	2.67	1.872	N/A

**Table 15:** RMSE results obtained in Example 5.

Filtration Conditions	CB	IB	SB	CF	EHM
First fouling stage (Uncoated membrane)	0.1122	0.04690	0.07190	0.05480	0.04190
Second fouling stage (Uncoated membrane)	0.1263	0.06240	0.08800	0.06690	0.05630
Third fouling stage (Uncoated membrane)	0.08860	0.06430	0.07590	0.04510	0.02730
First fouling stage (Coated membrane)	0.09500	0.04030	0.06220	0.04270	0.03400
Second fouling stage (Coated membrane)	0.1616	0.09510	0.1235	0.06880	0.06820
Third fouling stage (Coated membrane)	0.08090	0.05350	0.06610	0.03710	0.03390

**Table 16:** EHM parameters for the regressions obtained in Example 6 at 0.4 bar and different nanoparticle concentrations.

Filtration Conditions	P	K (s^−1^)	EHM Half-Life (s)
97 mgNPs/L	3.60	0.026	426.52
251 mgNPs/L	2.06	0.014	229.94
657 mgNPs/L	2.80	0.187	31.91

**Table 17:** RMSE results obtained in Example 6 at 0.4 bar and different nanoparticle concentrations.

Filtration Conditions	CB	IB	SB	CF	EHM
97 mgNPs/L	0.1271	0.0806	0.1004	0.0552	0.0425
251 mgNPs/L	0.1276	0.0609	0.0908	0.0294	0.0292
657 mgNPs/L	0.1480	0.0822	0.1124	0.0398	0.0288

## Data Availability

Not applicable.

## Appendix A

**Proof of Theorem** **1.** Taking all the equations presented in Section 2, it is possible to take the control volume from Section 2.1 and apply the continuity equation (Equation (32)). By assuming that the system has uniform entrances and exits, the surface integral can be reduced to:

(A1) $$\underset{CS}{\iint }\rho \vec{j}\cdot d\vec{A}=\underset{CS}{\sum }\rho_{i}j_{i}A_{i}$$

For the present system, there are two sources of flux: the entrance and the exit. As a result, this sum is given by:

(A2) $$\underset{CS}{\sum }\rho_{i}j_{i}A_{i}=\rho_{exit}j\left(t\right)A_{exit}-\rho_{ent}j_{0}A_{ent}$$

By the definition of flux given in Equation (36), $N_{0}=\rho_{in}j_{0}$ and $N\left(t\right)=\rho_{out}j\left(t\right)$. Since the control volume has a constant area, $A_{exit}=A_{ent}=A$. Therefore:

(A3) $$\underset{CS}{\sum }\rho_{i}j_{i}A_{i}=A\cdot \left(N\left(t\right)-N_{0}\right)$$

Thus, the surface integral of the Continuity Equation is simplified to:

(A4) $$\underset{CS}{\iint }\rho \vec{j}\cdot d\vec{A}=A\cdot \left(N\left(t\right)-N_{0}\right)$$

As for the volume integral, the control volume itself is a porous solid with a constant density $\rho_{s}$, a base area A and thickness $\delta \left(t\right)$:

(A5) $$\underset{CV}{∭}\rho d\forall =\rho_{s}\forall =\rho_{s}\cdot A\cdot \delta \left(t\right)$$

So:

(A6) $$\frac{\partial }{\partial t}\underset{CV}{∭}\rho d\forall =\rho_{s}\cdot A\cdot \frac{d\delta \left(t\right)}{dt}$$

Going back to the Continuity Equation:

$$\rho_{s}\cdot A\cdot \frac{d\delta \left(t\right)}{dt}+A\cdot \left(N\left(t\right)-N_{0}\right)=0\;$$

If $k_{1}=\rho_{s}$, then:

(A7) $$N\left(t\right)=N_{0}-k_{1}\cdot \frac{d\delta \left(t\right)}{dt}$$

According to Equation (33), for a real constant $k_{2}$:

(A8) $$\frac{d^{2}t}{dV^{2}}=k_{2}{\left(\frac{dt}{dV}\right)}^{n}$$

Using the first property presented in Equation (34), it is possible to write dt/dV in terms of dV/dt. Therefore:

(A9) $${\left(\frac{dt}{dV}\right)}_{V^{*}}={\left[{\left(\frac{dV}{dt}\right)}_{t^{*}}\right]}^{-1}$$

For $V\left(t^{*}\right)=V^{*}$. Now, using the second property from the same Section, it is possible to write $d^{2}t/dV^{2}$ in terms of $dV^{2}/dt^{2}$. Thus, Equation (A8) can be rewritten as:

$${\left(\frac{d^{2}t}{dV^{2}}\right)}_{V^{*}}=-{\left(\frac{d^{2}V}{dt^{2}}\right)}_{t^{*}}\cdot {\left[{\left(\frac{dt}{dV}\right)}_{V^{*}}\right]}^{3}\;$$

Using Equation (A9):

(A10) $${\left(\frac{d^{2}t}{dV^{2}}\right)}_{V^{*}}=-{\left(\frac{d^{2}V}{dt^{2}}\right)}_{t^{*}}\cdot {\left[{\left(\frac{dV}{dt}\right)}_{t^{*}}\right]}^{-3}$$

Applying Equations (A9) and (A10) to the Hermia model:

$$\left[-{\left(\frac{d^{2}V}{dt^{2}}\right)}_{t^{*}}\cdot {\left[{\left(\frac{dV}{dt}\right)}_{t^{*}}\right]}^{-3}\right]=k_{2}\cdot {\left\{{\left[{\left(\frac{dV}{dt}\right)}_{t^{*}}\right]}^{-1}\right\}}^{n}$$

$${\left(\frac{d^{2}V}{dt^{2}}\right)}_{t^{*}}=k_{3}{\left[{\left(\frac{dV}{dt}\right)}_{t^{*}}\right]}^{3-n}$$

If m=3−n and $k_{3}$ is another real constant, then:

$${\left(\frac{d^{2}V}{dt^{2}}\right)}_{t^{*}}=k_{3}{\left[{\left(\frac{dV}{dt}\right)}_{t^{*}}\right]}^{m}\;$$

Since both of these derivatives have the same domain of t, then for any $t^{*}$, this ODE is valid; therefore, it is possible to remove the subscript.

(A11) $$\frac{d^{2}V}{dt^{2}}=k_{3}{\left[\left(\frac{dV}{dt}\right)\right]}^{m}$$

It is important to note that both Equations (A8) and (A11) are analogous, which means that both functions $t\left(V\right)$ and $V\left(t\right)$ are solutions of the same family of differential equations. By the definition of accumulated volume presented in Equation (37), it is possible to use Equation (A7) such that;

$$V\left(t\right)=\frac{A}{\rho_{exit}}\int_{0}^{t}\left[N_{0}-k_{1}\frac{d\delta \left(t\right)}{dt}\right]dt$$

By the integral property presented in Equation (38):

$$\int_{0}^{t}\left[\frac{d\delta \left(t\right)}{dt}\right]dt=\delta \left(t\right)-\delta \left(0\right)\;$$

Since there is no mass accumulated in the control volume at t=0, then $\delta \left(0\right)=0$. As a result:

(A12) $$V\left(t\right)=\frac{A}{\rho_{exit}}\left[N_{0}\cdot t-k_{1}\delta \left(t\right)\right]$$

Differentiating $V\left(t\right)$ twice:

(A13) $$\frac{dV}{dt}=\frac{A}{\rho_{exit}}\left[N_{0}-k_{1}\frac{d\delta \left(t\right)}{dt}\right]$$

(A14) $$\frac{d^{2}V}{dt^{2}}=-k_{1}\frac{A}{\rho_{exit}}\cdot \frac{d^{2}\delta \left(t\right)}{dt^{2}}$$

With these derivatives, it is possible to rewrite Equation (A11) such that:

$$-k_{1}\frac{A}{\rho_{l}}\cdot \frac{d^{2}\delta \left(t\right)}{dt^{2}}=k_{3}{\left[\frac{A}{\rho_{exit}}\left[N_{0}-k_{1}\frac{d\delta \left(t\right)}{dt}\right]\right]}^{m}$$

For another real constant $k_{4}$:

(A15) $$\frac{d^{2}\delta \left(t\right)}{dt^{2}}=k_{4}{\left[N_{0}-k_{1}\frac{d\delta \left(t\right)}{dt}\right]}^{m}$$

Reducing Equation (A15) further with Equation (A7):

(A16) $$\frac{d^{2}\delta \left(t\right)}{dt^{2}}=k_{4}{\left[N\left(t\right)\right]}^{m}$$

By differentiating Equation (A7):

(A17) $$\frac{dN\left(t\right)}{dt}=-k_{1}\frac{d^{2}\delta \left(t\right)}{dt^{2}}$$

Therefore, Equation (A16) can be simplified further to:

(A18) $$\frac{dN\left(t\right)}{dt}=k_{5}{\left[N\left(t\right)\right]}^{m}$$

such that $k_{5}$ is another real constant. Now, by applying the separation of variables method [17]:

$${\left[N\left(t\right)\right]}^{-m}dN\left(t\right)=k_{5}dt$$

As a result, for m≠1:

$$\int_{N\left(0\right)}^{N\left(t\right)}{\left[N\left(t\right)\right]}^{-m}dN\left(t\right)=\int_{0}^{t}k_{5}dt$$

$$\left[\frac{{\left[N\left(t\right)\right]}^{\left(1-m\right)}}{\left(1-m\right)}\right]\begin{array}{c} N\left(t\right)\\ N\left(0\right) \end{array}=\left[k_{5}t\right]\begin{array}{c} t\\ 0 \end{array}$$

$$\frac{1}{\left(1-m\right)}\left\{{\left[N\left(t\right)\right]}^{1-m}-{\left[N\left(0\right)\right]}^{1-m}\right\}=k_{5}t$$

If $P=-\left(1-m\right)$:

$${\left[N\left(t\right)\right]}^{-P}={\left[N\left(0\right)\right]}^{-P}-P\cdot k_{5}\cdot t$$

and $-Pk_{5}=k_{6}$:

$${\left[N\left(t\right)\right]}^{-P}={\left[N\left(0\right)\right]}^{-P}+k_{6}\cdot t$$

Since there is no mass accumulated at the beginning of the filtration experiment ($\delta \left(0\right)=0$), both $N_{0}$ and $N\left(0\right)$ are equal. Therefore:

(A19) $${\left[N\left(t\right)\right]}^{-P}={\left[N_{0}\right]}^{-P}+k_{6}\cdot t$$

It is important to notice that Equation (30) is similar to the original equations used in the Hermia model. It is also important to highlight that both P and $k_{6}$ can assume any real values, as long as P≠0. By using the flux definition presented in Section 2.4:

$${\left[\rho_{exit}j\left(t\right)\right]}^{-P}={\left[\rho_{ent}j_{0}\right]}^{-P}+k_{6}\cdot t$$

Thus, if $k_{7}$ is another real constant:

(A20) $${\left[j\left(t\right)\right]}^{-P}={\left[\frac{\rho_{ent}}{\rho_{exit}}\right]}^{-P}\cdot j_{0}^{-P}+k_{7}\cdot t$$

As a result, Equation (31) closely resembles the power law used in the Hermia fouling model. However, the additional term ${\left[\rho_{ent}/\rho_{exit}\right]}^{-P}\;$ correctly scales up $j_{0}$ such that the right-hand side of Equation (31) agrees with the Continuity Equation. In special cases where the density of the permeate is close to the original density ($\rho_{exit}\to \rho_{ent}$), the correction term ${\left[\rho_{ent}/\rho_{exit}\right]}^{-P}\to 1$. If that is the case, ${\left[j\left(t\right)\right]}^{-P}$ can be approximated by:

$${\left[j\left(t\right)\right]}^{-P}\;\approx j_{0}^{-P}+k_{7}\cdot t$$

Therefore, for a new constant k:

(A21) $${\left[\frac{j\left(t\right)}{j_{0}}\right]}^{P}\approx \frac{1}{\left(1+k\cdot t\right)}$$

For the special case when m=1:

$$\int_{N_{0}}^{N\left(t\right)}\frac{1}{N\left(t\right)}dN\left(t\right)=\int_{0}^{t}k_{5}dt$$

$$\ln\left[\frac{N\left(t\right)}{N_{0}}\right]=k_{5}t$$

$$N\left(t\right)=N_{0}\exp\left(k_{5}t\right)$$

If $k=-k_{5}$, then:

(A22) $$N\left(t\right)=N_{0}\exp\left(-k\cdot t\right)$$

Since Equation (A22) has an exponential function multiplying $N_{0}$, and k can assume positive values (or $k_{5}<0$), when m=1, the system can behave with a classical drop for $N\left(t\right)$. Applying again the flux definition presented in Equation (36):

$$\rho_{exit}j\left(t\right)=\rho_{ent}j_{0}\exp\left(-k\cdot t\right)\;$$

(A23) $$j\left(t\right)=j_{0}\left[\frac{\rho_{ent}}{\rho_{exit}}\right]\exp\left(-k\cdot t\right)$$

For the same reasons as previously specified, if $\rho_{exit}\to \rho_{ent}$, $\left[\rho_{ent}/\rho_{exit}\right]\to 1\;$ and $j\left(t\right)$ can be approximated by:

(A24) $$j\left(t\right)\approx j_{0}\exp\left(-k\cdot t\right)$$

It is possible to conclude that P=2−n. By using the four original discrete values of n=2, 1,3/2,0, P=0,1,1/2,2, which are exactly the respective exponents of j in Equations (1)–(4). Thus, through Equations (A21) and (A24), it is possible to reproduce the entire Hermia model. Since Equation (A21) was obtained for any real n≠2  and Equation (A24) was obtained for n=2, these equations form a model that can be used for any real n, which widens the usefulness of the Hermia model considerably. Consequently, there are also values of n between the four original discrete values, which can be physically interpreted as the existence of new types of fouling mechanisms in membranes. □

## Appendix B

**Proof of Theorem** **2.** It is also possible to deduce how the fouling profile should change with P, given by the function $\delta \left(t\right)$. Through Equations (9) and (18), it is possible to show that:

(A25) $$\left[\frac{j\left(t\right)}{j_{0}}\right]=\left[\frac{\rho_{ent}}{\rho_{exit}}\right]-k_{10}\frac{d\delta \left(t\right)}{dt}$$

For another real constant $k_{10}$. For the case of P≠0, the profile of $j\left(t\right)$ is given by Equation (A20). Taking this equation and dividing both sides by $j_{0}^{-P}$:

(A26) $${\left[\frac{j\left(t\right)}{j_{0}}\right]}^{-P}={\left[\frac{\rho_{ent}}{\rho_{exit}}\right]}^{-P}+k_{7}t$$

Since $k_{7}/j_{0}^{-P}$ is still a constant, the same constant will be used. Therefore, by substituting Equation (37), into Equation (A25) into Equation (A26):

$${\left[\left[\frac{\rho_{ent}}{\rho_{exit}}\right]-k_{10}\frac{d\delta \left(t\right)}{dt}\right]}^{-P}={\left[\frac{\rho_{ent}}{\rho_{exit}}\right]}^{-P}+k_{7}t\;$$

$$\left[\frac{\rho_{ent}}{\rho_{exit}}\right]-k_{10}\frac{d\delta \left(t\right)}{dt}={\left({\left[\frac{\rho_{ent}}{\rho_{exit}}\right]}^{-P}+k_{7}t\right)}^{-1/P}\;$$

$$-k_{10}\frac{d\delta \left(t\right)}{dt}={\left({\left[\frac{\rho_{ent}}{\rho_{exit}}\right]}^{-P}+k_{7}t\right)}^{-\frac{1}{P}}-\left[\frac{\rho_{ent}}{\rho_{exit}}\right]\;$$

If $k_{11}$ and $k_{12}$ are other real constants, then:

$$\frac{d\delta \left(t\right)}{dt}=k_{11}{\left({\left[\frac{\rho_{ent}}{\rho_{exit}}\right]}^{-P}+k_{7}t\right)}^{-\frac{1}{P}}+k_{12}\;$$

$$\frac{d\delta \left(t\right)}{dt}={\left(k_{11}^{-P}{\left[\frac{\rho_{ent}}{\rho_{exit}}\right]}^{-P}+k_{11}^{-P}k_{7}t\right)}^{-\frac{1}{P}}+k_{12}\;$$

Thus, if $k_{13}=k_{11}^{-P}{\left[\rho_{ent}/\rho_{exit}\right]}^{-P}$ and $k_{14}=k_{11}^{-P}k_{7}$:

(A27) $$\frac{d\delta \left(t\right)}{dt}={\left(k_{13}+k_{14}t\right)}^{-\frac{1}{P}}+k_{12}$$

Now, by applying the separation of variables method in Equation (A27):

$$\int_{\delta \left(0\right)}^{\delta \left(t\right)}d\delta \left(t\right)=\int_{0}^{t}[{\left(k_{13}+k_{14}t\right)}^{-\frac{1}{P}}+k_{12}]dt\;$$

Since:

$$\int_{\delta \left(0\right)}^{\delta \left(t\right)}d\delta \left(t\right)=\delta \left(t\right)-\delta \left(0\right)\;$$

And $\delta \left(0\right)=0$, then:

(A28) $$\delta \left(t\right)=\int_{0}^{t}[{\left(k_{13}+k_{14}t\right)}^{-\frac{1}{P}}+k_{12}]dt$$

By integrating Equation (39) with respect to t:

$$\delta \left(t\right)=\left[\frac{1}{k_{14}}\frac{{\left(k_{13}+k_{14}t\right)}^{-\frac{1}{P}+1}}{\left(-\frac{1}{P}+1\right)}+k_{12}t\right]\begin{array}{c} t\\ 0 \end{array}$$

$$\delta \left(t\right)=\frac{1}{k_{14}}\left[\frac{{\left(k_{13}+k_{14}t\right)}^{-\frac{1}{P}+1}-k_{13}^{-\frac{1}{P}+1}}{\left(-\frac{1}{P}+1\right)}\right]+k_{12}t\;$$

It is possible to further reduce this equation by distributing the terms $1/k_{14}$ and $\left(-\frac{1}{P}+1\right)$. By doing this, it should be possible to regroup the constants inside the brackets of ${\left(k_{13}+k_{14}t\right)}^{-\frac{1}{P}+1}$. If:

$$k_{15}=\frac{k_{13}}{{\left(k_{14}\cdot \left(-\frac{1}{P}+1\right)\right)}^{-\frac{1}{P}+1}}\;$$

$$k_{16}=\frac{k_{14}}{{\left(k_{14}\cdot \left(-\frac{1}{P}+1\right)\right)}^{-\frac{1}{P}+1}}\;$$

$$k_{17}=\frac{-k_{13}^{-\frac{1}{P}+1}}{k_{14}\cdot \left(-\frac{1}{P}+1\right)}\;$$

Then:

(A29) $$\delta \left(t\right)={\left(k_{15}+k_{16}t\right)}^{-\frac{1}{P}+1}+k_{17}+k_{12}t$$

By applying $\delta \left(0\right)=0$ in Equation (A29), it can be found that:

$$k_{17}=-k_{15}^{-\frac{1}{P}+1}\;$$

As a result, Equation (A29) can be further reduced to:

(A30) $$\delta \left(t\right)={\left(k_{15}+k_{16}t\right)}^{-\frac{1}{P}+1}-k_{15}^{-\frac{1}{P}+1}+k_{12}t$$

For the case of P=0, through Equations (A24) and (A25), it is possible to show that:

$$\left[\frac{\rho_{ent}}{\rho_{exit}}\right]\exp\left(-k_{9}t\right)=\left[\frac{\rho_{ent}}{\rho_{exit}}\right]-k_{10}\frac{d\delta \left(t\right)}{dt}\;$$

Dividing both sides by $\left[\rho_{ent}/\rho_{exit}\right]$:

$$\exp\left(-k_{9}t\right)=1-k_{10}\left[\frac{\rho_{exit}}{\rho_{ent}}\right]\frac{d\delta \left(t\right)}{dt}\;$$

If $1/k_{18}=-k_{10}\left[\rho_{exit}/\rho_{ent}\right]$, then:

$$\exp\left(-k_{9}t\right)=1+\frac{1}{k_{18}}\frac{d\delta \left(t\right)}{dt}\;$$

Therefore:

(A31) $$\frac{d\delta \left(t\right)}{dt}=k_{18}\left[\exp\left(-k_{9}t\right)-1\right]$$

Now, by applying the separation of variables method in Equation (A31) [16]:

$$\int_{\delta \left(0\right)}^{\delta \left(t\right)}d\delta \left(t\right)=k_{18}\int_{0}^{t}\left[\exp\left(-k_{9}t\right)-1\right]dt\;$$

$$\delta \left(t\right)=k_{18}\left[\frac{1}{-k_{9}}\exp\left(-k_{9}t\right)-t\right]\begin{array}{c} t\\ 0 \end{array}\;$$

$$\delta \left(t\right)=-\frac{k_{18}}{k_{9}}\left[\exp\left(-k_{9}t\right)-1\right]-k_{18}t\;$$

If $k_{19}=-k_{18}/k_{9}$ and $k_{20}=-k_{18}$, then:

(A32) $$\delta \left(t\right)=k_{19}\left[\exp\left(-k_{9}t\right)-1\right]+k_{20}t$$

As a result, with Equations (A29) and (A32), it is possible to construct a fouling model for every real value of P, such that:

(A33) δt=k15+k16t−1P+1−k15−1P+1+k12t,  P≠0 k19exp−k9t−1+k20t,  P=0

It is important to notice that the different fouling profiles δ are given by the exponent −1/P+1. Therefore, by analyzing this exponent, it is possible to conclude the fouling profiles as well. By taking the limit as P→∞,−1/P→0:

$$\delta \left(t\right)={\left(k_{15}+k_{16}t\right)}^{0+1}-k_{15}^{0+1}+k_{12}t$$

$$\delta \left(t\right)=\left(k_{16}+k_{12}\right)t$$

Now, by taking the limit as P→−∞, −1/P→0:

$$\delta \left(t\right)={\left(k_{15}+k_{16}t\right)}^{0+1}-k_{15}^{0+1}+k_{12}t\;$$

$$\delta \left(t\right)=\left(k_{16}+k_{12}\right)t\;$$

Therefore, for both P→∞ and P→−∞, the result is a linear curve. The same behavior can be seen when P=1, since:

$$\delta \left(t\right)={\left(k_{15}+k_{16}t\right)}^{1-1}-k_{15}^{1-1}+k_{12}t$$

$$\delta \left(t\right)=k_{12}t$$

For positive values of P, the exponent −1/P+1 is always smaller than 1; in contrast, for negative values of P, −1/P+1 is always larger than 1. Consequently, there is one and only one unique fouling profile $\delta \left(t\right)$ for every real value P. □

## Appendix C

**Proof of Theorem** **3.**

$$\frac{dV}{dt}=j\left(t\right)\cdot A$$

$$dV=j\left(t\right)\cdot A\cdot dt$$

For P≠0:

$${\left[\frac{j\left(t\right)}{j_{0}}\right]}^{P}\approx \frac{1}{\left(1+k\cdot t\right)}$$

Therefore:

$$dV=j_{0}\cdot A\cdot {\left[\frac{1}{\left(1+k\cdot t\right)}\right]}^{\frac{1}{P}}\cdot dt$$

$$\overset{V\left(t\right)}{\underset{V\left(0\right)}{\int }}dV=j_{0}\cdot A\cdot \overset{t}{\underset{0}{\int }}{\left[\frac{1}{\left(1+k\cdot t\right)}\right]}^{\frac{1}{P}}\cdot dt$$

$$V\left(t\right)-V\left(0\right)=j_{0}\cdot A\cdot \overset{t}{\underset{0}{\int }}{\left[\left(1+k\cdot t\right)\right]}^{-\frac{1}{P}}\cdot dt$$

Since there is no permeate at t=0, $V\left(0\right)=0$:

$$V\left(t\right)=j_{0}\cdot A\cdot \left[\frac{{\left[\left(1+k\cdot t\right)\right]}^{1-\frac{1}{P}}}{k\cdot \left(1-\frac{1}{P}\right)}\right]\begin{array}{c} t\\ 0 \end{array}$$

$$V\left(t\right)=\frac{j_{0}\cdot A}{k\left(1-\frac{1}{P}\right)}({\left[1+k\cdot t\right]}^{\left(1-\frac{1}{P}\right)}-1)$$

For P=0:

$$\frac{j\left(t\right)}{j_{0}}\approx \exp\left(-k\cdot t\right)$$

Thus:

$$\overset{V\left(t\right)}{\underset{V\left(0\right)}{\int }}dV=j_{0}\cdot A\cdot \overset{t}{\underset{0}{\int }}\exp\left(-k\cdot t\right)\cdot dt$$

$$V\left(t\right)=j_{0}\cdot A\cdot \left[-\frac{\exp\left(-k\cdot t\right)}{k}\right]\begin{array}{c} t\\ 0 \end{array}$$

$$V\left(t\right)=\frac{j_{0}\cdot A}{k}\cdot \left[1-\exp\left(-k\cdot t\right)\right]$$

Therefore:

$$V\left(t\right)=\left\{\begin{array}{c} \frac{j_{0}\cdot A}{k\left(1-\frac{1}{P}\right)}({\left[1+k\cdot t\right]}^{\left(1-\frac{1}{P}\right)}-1)\;,\;\;P\neq 0\\ \frac{j_{0}\cdot A}{k}\left[1-\exp\left(-k\cdot t\right)\right],\;\;P=0\; \end{array}\right.$$

□

## Appendix D. Complementary Figures

Example 1:

Example 2:

Example 3:

Example 4:

Example 5:

Example 6:
